# Picosecond reactions of excited radical ion super-reductants

**DOI:** 10.1038/s41467-024-49006-5

**Published:** 2024-06-04

**Authors:** Björn Pfund, Deyanira Gejsnæs-Schaad, Bruno Lazarevski, Oliver S. Wenger

**Affiliations:** Department of Chemistry, University of Basel, Basel, Switzerland

**Keywords:** Photocatalysis, Photocatalysis

## Abstract

Classical photochemistry requires nanosecond excited-state lifetimes for diffusion-controlled reactions. Excited radicals with picosecond lifetimes have been implied by numerous photoredox studies, and controversy has arisen as to whether they can actually be catalytically active. We provide direct evidence for the elusive pre-association between radical ions and substrate molecules, enabling photoinduced electron transfer beyond the diffusion limit. A strategy based on two distinct light absorbers, mimicking the natural photosystems I and II, is used to generate excited radicals, unleashing extreme reduction power and activating C(sp^2^)―Cl and C(sp^2^)―F bonds. Our findings provide a long-sought mechanistic understanding for many previous synthetically-oriented works and permit more rational future photoredox reaction development. The newly developed excitation strategy pushes the current limits of reactions based on multi-photon excitation and very short-lived but highly redox active species.

## Abstract (web summary)

Classical photochemistry requires nanosecond excited-state lifetimes for diffusion-controlled reactions. Here the authors provide direct evidence for the elusive pre-association between radical ions and substrate molecules, enabling photoinduced electron transfer beyond the diffusion limit.

## Introduction

**Fig. 1:**
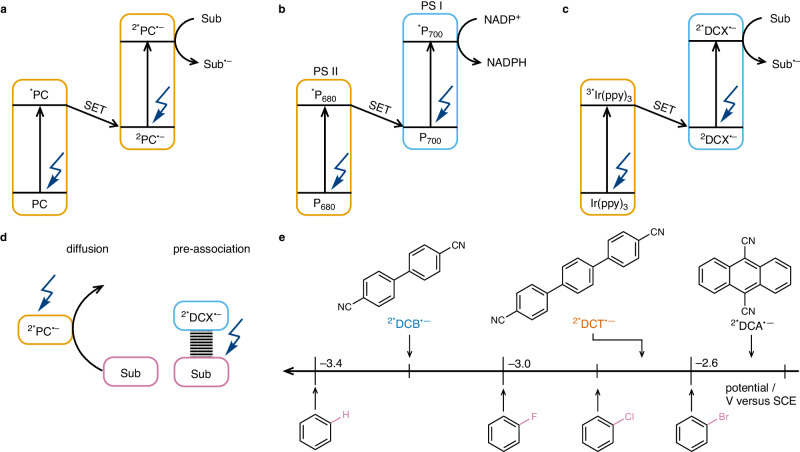
Two-photon excitation strategies to form super-reductants. **a** Previously demonstrated strategy termed consecutive photoinduced electron transfer (ConPET), where a given molecule acts both as a light-harvesting photosensitizer and as a photocatalyst in its radical anion form (PC^•–^)^8^. **b** Natural Z-scheme excitation strategy with the two key chromophores of photosystem II (PS II, P680) and photosystem I (PS I, P700). **c** New strategy of sensitized ConPET, combining Ir(ppy)_3_ as a light-harvesting photosensitizer and dicyanoarene radical anions (DCX^•–^) as the redox-active photocatalysts^27^. The first photon promotes Ir(ppy)_3_ to its lowest triplet excited state (^3*^Ir(ppy)_3_), which reduces the dicyanoarenes to their radical anion forms (DCX^•–^) via single electron transfer (SET). The second photon excites DCX^•–^, forming the strongly reducing excited radical anion (^2*^DCX^•–^). **d** Classical photoredox reactions rely on bimolecular diffusion to bring the substrate (Sub) to reaction with the PC, typically requiring excited-state lifetimes in the nanosecond regime or longer^21^. Pre-association between Sub and PC^•–^ can enable photoreactivity within the picosecond excited state lifetimes of organic radicals. **e** Reduction potentials of some benchmark substrates^46–49^ and the excited radical anions of 9,10-dicyanoanthracene (^2*^DCA^•–^)^9,27^, 4,4″-dicyano-*p*-terphenyl (^2*^DCT^•–^), and 4,4′-dicyanobiphenyl (^2*^DCB^•–^).

Single electron transfer (SET) is the key elementary step in photoredox catalysis, enabling many chemical transformations under mild conditions^1–3^. The energy of visible light usually limits the accessible redox potential to a rather narrow range, restricting the scope of reactivity^4^. Electronically excited organic radicals have been claimed to surpass this limitation^5–14^. However, whether excited radical ions can truly act as catalytically active species has remained contentious, in some cases due to the formation of photoactive degradation products^15–17^, but generally owing to the picosecond lifetimes of the radical ion excited states^18–21^, which are too short for diffusion-based SET (Fig. 1d)^22^. Therefore, pre-association between the radical ions with substrate molecules prior to electronic excitation is commonly thought to account for the observable photoreactivity^12,13,15^. The lack of direct evidence for pre-association and the missing clear-cut identification of excited radical ions as catalytically active species limits the rational design of photoredox and electro-photoredox catalysis targeting extreme potentials^23–26^.

Additional limitations arise from the fact that the common excitation strategy for consecutive photoinduced electron transfer (ConPET, Fig. 1a) imposes a dual role on the supposed catalytically active species^27^. In known ConPET systems, a precursor molecule absorbs the first photon and is converted into a radical ion through photoinduced SET. The radical ion is then excited by a second photon to form the presumed photocatalyst. This excitation strategy restricts the maximally achievable efficiency of the overall process and imposes thermodynamic limitations that restrict reaction scopes. To overcome these shortcomings, we report an excitation strategy inspired by natural photosynthesis, in which two different chromophores cooperate (Fig. 1b). In this sensitized ConPET approach (Fig. 1c), a photosensitizer (PS) first absorbs a photon and undergoes SET with the photocatalyst precursor, forming a radical anion that is subsequently excited with a second photon. The two distinct catalytic cycles resulting from this Z-scheme approach provide greater control over light absorption and SET kinetics and can, therefore, enhance the overall efficiency of the process. This new strategy provides access to very strongly reducing species that formerly were inaccessible with visible light.

Starting from the widely used 9,10-dicyanoanthracenyl radical anion (DCA^•–^, Fig. 1e)^5,9,27^, we identified 4,4”-dicyano-*p*-terphenyl (DCT, Fig. 1e) and 4,4′-dicyanobiphenyl (DCB, Fig. 1e) as potentially even stronger reductants in their excited radical anionic forms. With the known ConPET strategy (Fig. 1a), excited DCT^•–^ and DCB^•–^ were inaccessible using visible light because DCT and DCB only absorb in the ultraviolet. The sensitized ConPET strategy (Fig. 1c) uses blue light for photoinduced SET from Ir(ppy)_3_ (Hppy, 2-phenylpyridine) to form DCT^•–^ and DCB^•–^. Upon visible light excitation of these radical anions, the activation of C(sp^2^)―Cl and C(sp^2^)―F bonds, requiring potentials up to –3.0 V versus SCE, was achieved (Fig. 1e). Despite the radical anion’s very short excited state lifetimes between 1 and 4 ps, two-color pump-pump-probe laser flash photolysis provides evidence for rapid SET, indicating pre-association. Ultra-fast transient UV-Vis absorption spectroscopy gives quantitative insight into the long-sought pre-association between radical anions and substrate molecules.

## Results

### Establishing the working principle of sensitized conPET

**Fig. 2:**
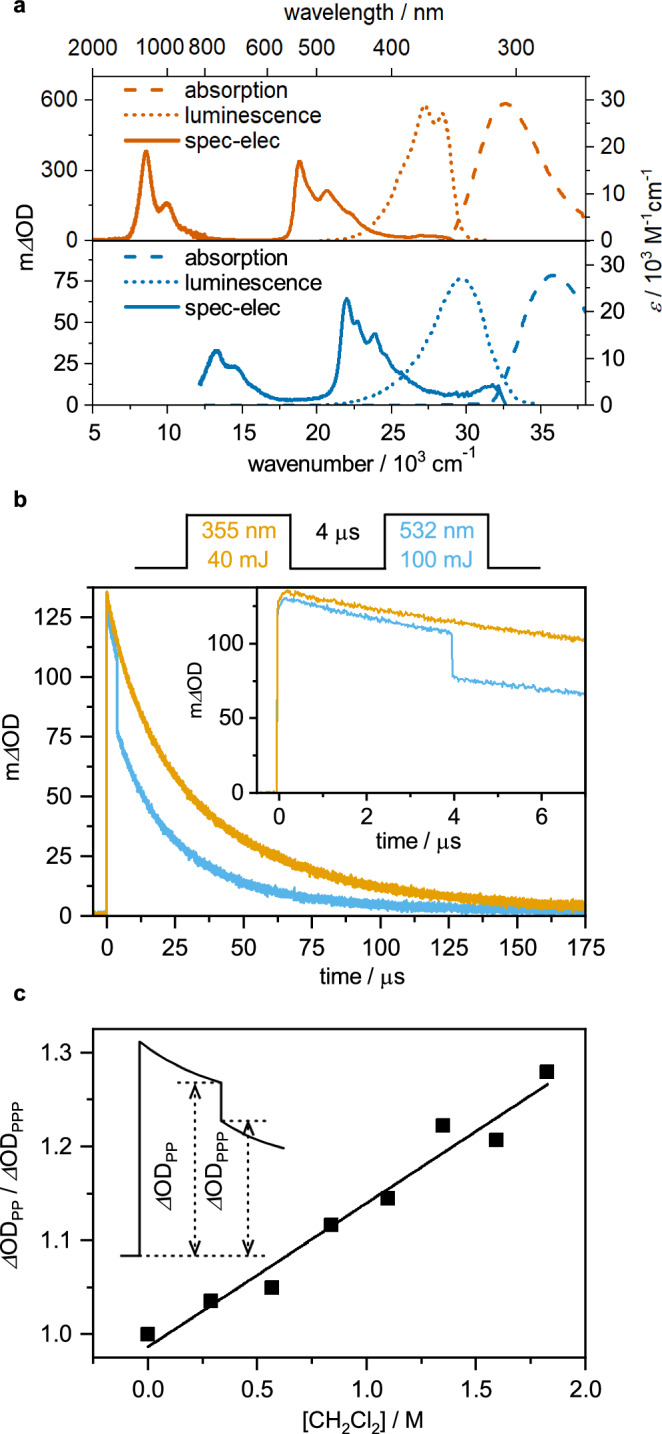
Basic spectroscopy and photoinduced electron transfer. **a** UV-Vis absorption spectra, normalized luminescence spectra upon excitation at 320 nm (DCT) and 290 nm (DCB), and absorption changes following electrochemical one-electron reduction of DCX at 20 °C. **b** Transient absorption kinetics for DCT^•–^ at 500 nm in a two-color pump-pump-probe experiment. The first laser pulse (355 nm, 40 mJ) generated DCT^•–^, whereas a second laser pulse (532 nm, 100 mJ) selectively excited DCT^•–^. An argon-saturated DMF solution containing 2 mM DCT, 200 mM DMA, and 280 mM CH_2_Cl_2_ at 20 °C was used. **c** Stern-Volmer plot based on the two-color pump-pump-probe experiment performed at variable CH_2_Cl_2_ concentrations. Inset: illustration of the two key observables ΔOD_PP_ (PP = pump-probe, monitored change in optical density immediately before the second pump pulse) and ΔOD_PPP_ (PPP = pump-pump-probe, monitored change in optical density immediately after the second pump pulse).

Based on cyclic voltammetry, the one-electron reduction potentials (*E*_red_) are –1.7 V for DCT and –1.6 V versus saturated calomel electrode (SCE) for DCB (Supplementary Fig. 2). DCT^•–^ and DCB^•–^ absorb in the visible and near-infrared (Fig. 2a)^28^ owing to electronic transitions from the D_0_ ground state to D_1_ and D_2_ excited states^19,29^. The D_1_ energies (*E*_D1_) are 1.0 eV for ^2*^DCT^•–^ and 1.6 eV for ^2*^DCB^•–^ (Supplementary Table 1), leading to excited state reduction potentials ^2*^E_red_ of ~–2.7 V versus SCE for ^2*^DCT^•–^ and –3.2 V versus SCE for ^2*^DCB^•–^ (Fig. 1e).

**Fig. 3:**
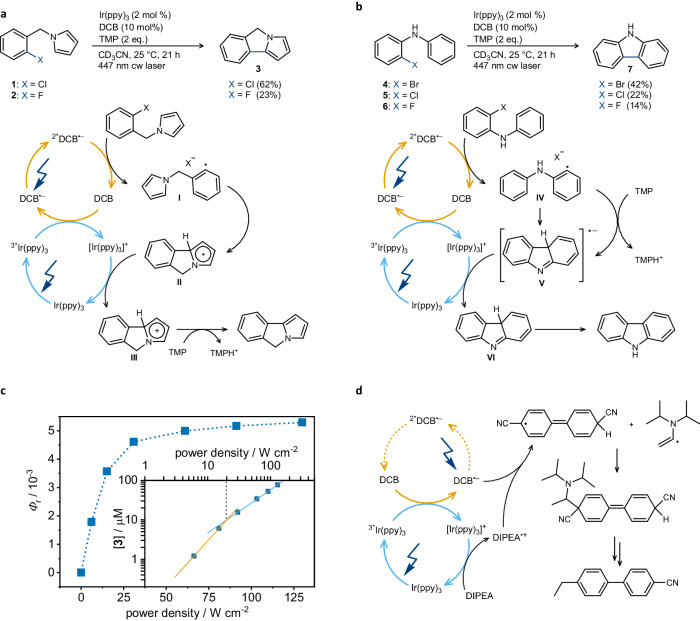
Photocatalytic reductive dehalogenations via sensitized ConPET, power dependence, and photo-degradation. **a** Base-promoted homolytic aromatic substitution (BHAS) of aryl halides along with a plausible mechanism. **b** Intra-molecular radical nucleophilic substitution reaction of 2-halide-*N*-phenylanilines to carbazole along with a plausible mechanism. Product yields were determined by ^1^H-NMR spectroscopy using trimethyl(phenyl)silane as internal standard. **c** Initial product formation quantum yield (determined after 30 minutes of irradiation) for the reaction of **1** to **3** at different excitation power densities at 447 nm. Inset: double logarithmic plot of the product concentration reached after 30 minutes as a function of excitation power density. **d** Photodecomposition of DCB upon 447 nm cw laser (1 W) irradiation of a solution containing Ir(ppy)_3_ (2 mM), DCB (20 mM), and DIPEA (200 mM) in argon-saturated CD_3_CN. In the overall redox-neutral reactions in **a** and **b**, no tertiary amine donors are involved, and the Ir(ppy)_3_/DCB system is more robust under these conditions.

The reactivity of the excited organic radicals was first probed using nanosecond two-color pump-pump-probe spectroscopy (Fig. 2b)^18,30^. After direct excitation of DCT with 355 nm pulses in the presence of excess *N*,*N*-dimethylaniline (DMA), DCT^•–^ is generated by SET from DMA (Supplementary Fig. 3). After a delay of 4 μs following the 355 nm laser pulse, a second laser pulse selectively excited DCT^•–^ at 532 nm, forming ^2*^DCT^•–^. In DMF without any electron accepting molecules added, the 532 nm laser pulse has no detectable influence on the DCT^•–^ transient absorption signal (Supplementary Fig. 4), because photo-excited DCT^•–^ decays back to its ground state within the 10 ns duration of the laser pulse^19^. However, in the presence of an electron-acceptor such as CH_2_Cl_2_ (Fig. 2b) or *α*,*α*,*α*-trifluorotoluene (Supplementary Fig. 5), the 532 nm pulse causes an instant bleach of the DCT^•–^ absorbance at 500 nm, indicating the immediate disappearance of DCT^•–^ (Fig. 2b, blue trace). This is attributable to SET from ^2*^DCT^•–^ to the electron acceptor, resulting in the instant formation of charge-neutral DCT, which does not absorb at 500 nm. Expectedly, an increase of the electron acceptor concentration leads to a stronger signal bleach upon the second laser pulse (Supplementary Fig. 6). The effect of the 532 nm pulse (with the specific energy of 100 mJ) is quantified by comparing the changes in optical densities (ΔOD) at 500 nm determined immediately before that pulse (named ΔOD_PP_; PP = pump-probe) and immediately afterward (named ΔOD_PPP_; PPP = pump-pump-probe)^30^. A plot of ΔOD_PP_ / ΔOD_PPP_ against the CH_2_Cl_2_ concentration (Fig. 3c) gives a rate constant of 1.1 × 10^10^ M^–1^ s^–1^ for SET from ^2*^DCT^•–^ to CH_2_Cl_2_ (Supplementary Fig. 6). This is 44% faster than the diffusion-limited reaction rate constant in DMF (7.6 × 10^9^ M^–1^ s^–1^) at 20 °C^31^, suggesting pre-association between DCT^•–^ and CH_2_Cl_2_. The formed SET products from CH_2_Cl_2_ or *α*,*α*,*α*-trifluorotoluene reduction do not have diagnostic UV-Vis signatures and cannot be monitored. DCB^•–^ absorbs very weakly at 532 nm, which hinders analogous two-color pump-pump-probe experiments, but the close chemical relationship with DCT suggests similar SET behavior in ^2*^DCB^•–^. In the above experiments, DCT was excited at 355 nm because this charge-neutral form does not absorb in the visible (Fig. 2a, dashed traces), but UV excitation is impractical for photocatalysis. Ir(ppy)_3_ sensitizes the formation of DCT^•–^ and DCB^•–^ with blue light. Photoinduced SET from Ir(ppy)_3_ to DCB occurs with 1.9 × 10^9 ^M^–1^ s^–1^ (Supplementary Fig. 8), whilst for DCT there are competing SET and triplet-triplet energy transfer (TTET) reactions (Supplementary Fig. 9). However, in the presence of DMA, only the DCT^•–^ photoproduct is observed, indicating SET from DMA to the triplet excited DCT. Hence, excitation of Ir(ppy)_3_ at 450 nm in the presence of excess electron donor leads to rapid and efficient formation of DCB^•–^ and DCT^•–^.

### Photocatalysis of aryl radical substitution reactions via reductive dehalogenations

ConPET strategies for photocatalysis typically rely on amine-based sacrificial electron donors such as DIPEA (*N*,*N*-diisopropylethylamine)^9,10^, forming PC^•–^ and an aminium radical cation (DIPEA^•+^) in the first step. Aminium radical cations and their deprotonated forms are highly reactive and can lead to unwanted side reactions, such as the decomposition of DCB forming 4′-ethyl-[1,1′-biphenyl]−4-carbonitrile (Fig. 3d), as observed in initial test reactions. The formation of degradation products with possible photo(redox) activity is problematic, as illustrated by the recent case of naphthalene monoimide (NMI). Initially suspected to react from its radical anion excited state^6^, a two-electron reduced (closed-shell) Meisenheimer complex [NMI(H)]^–^ was later identified as a perhaps more plausible photoactive substance^16^. Similarly, for the structurally related DCA^•–^ (Fig. 1e), excited state lifetimes >1 ns were initially reported^32^, but were later found to originate from degradation products^17^. Against this background, we focus on overall redox neutral reactions not requiring any sacrificial reductants. The base-promoted homolytic aromatic substitution (BHAS) reaction of 1-(2-chlorobenzyl)−1H-pyrrole (**1**) to 5H-pyrrolo[2,1-a]isoindole (**3**) was chosen^33,34^. This reaction requires a reduction potential of approximately –2.8 V versus SCE for reductive dechlorination, similar to chlorobenzene (Fig. 1e). Owing to the stronger reducing power of ^2*^DCB^•–^ (^2*^E_red_ = –3.2 V versus SCE), and the efficient SET from ^3*^Ir(ppy)_3_, DCB was preferred over DCT.

Using 2 mol% Ir(ppy)_3_, 10 mol% of DCB, and 2 equivalents of TMP (2,2,6,6-tetramethylpiperidine) in argon-saturated acetonitrile at 25 °C, product **3** was formed in 62% yield upon irradiation with continuous wave (cw) laser at 447 nm for 21 hours (Supplementary Fig. 12). 5% of the hydrodechlorination side product (1-benzylpyrrole) were formed (Supplementary Fig. 11), which together with the main product **3** can account for the observed conversion of 72%. Control experiments without Ir(ppy)_3_, DCB, TMP, or light did not result in product formation (Supplementary Table 2). The expectable mechanism for the BHAS reaction of substrate **1** (Fig. 3a) begins with photoinduced SET from Ir(ppy)_3_ to DCB forming DCB^•‒^ and [Ir(ppy)_3_]^+^^33,34^. Excitation of DCB^•‒^ then forms ^2*^DCB^•‒^, which is sufficiently reducing to activate **1** if pre-association between DCB^•‒^ and **1** occurs (a key aspect that will be addressed below). The radical aryl intermediate **I** formed after reductive dechlorination is intercepted intramolecularly by the pyrrole unit, resulting in **II**. Oxidation of **II** by [Ir(ppy)_3_]^+^ restores the initial form of the photosensitizer and leads to the radical cation **III**. Following deprotonation by TMP, the cyclization product **3** is formed. To demonstrate the versatility of our newly developed sensitized conPET strategy, several different iridium-based and organic photosensitizers with similar excited state reduction potentials were tested, resulting in comparable product formation (Supplementary Table 2). Hence there is no strict need for precious metal-based photosensitizers. Using identical reaction conditions compared to the aryl chloride **1**, the more difficult to reduce aryl fluoride **2** was converted to product **3** in 23% yield (Supplementary Fig. 14).

Aside from the BHAS reactions in Fig. 3a, the intra-molecular radical nucleophilic substitution reaction of 2-halide-*N*-phenylanilines (**4** - **6**) to carbazole (**7**) was investigated (Fig. 3b). Here, ^2*^DCB^•–^ is hypothesized to activate the aryl-halide bond to form intermediate **IV**, followed by a nucleophilic addition, generating intermediate **V**^33^. Oxidation of **V** by [Ir(ppy)_3_]^+^ restores Ir(ppy)_3_ and leads to intermediate **VI**, which upon proton transfer forms carbazole **7** (Fig. 3b). Under similar conditions as for the BHAS reactions, the aryl-bromide substrate (**4**) reacted to product **7** with a yield of 42% (Supplementary Fig. 15), whereas chloro-*N*-phenylamine (**5**) and fluoro-*N*-phenylamine (**6**) resulted in lower yields of 22% and 14%, respectively. The trend in decreasing carbazole yield along the series **4** > **5** > **6** reflects the increasingly difficult initial reductive dehalogenation step. The generally lower yields in Fig. 3b compared to the BHAS reactions could be due to the electron-rich nature of the aniline substrates, less efficient radical interception by phenyl than pyrrole^35^, and slower regeneration of Ir(ppy)_3_ by intermediate **V** compared to intermediate **II**^34^.

BHAS reactions can proceed via a radical chain mechanism^33^, hence the reaction quantum yield (*Φ*_*r*_) of **1** was investigated. During the first 30 minutes the *Φ*_*r*_ is ~5 × 10^–3^ (Supplementary Fig. 19)^36^, followed by a decrease over extended irradiation times due to photo-degradation and a *Φ*_*r*_ value of ~2 × 10^–3^ after 3 h. When the reaction mixture was alternately irradiated with light and then left in the dark (Supplementary Fig. 18), no product formation occurred during the dark periods. Collectively, these experiments signal the absence of a radical chain mechanism^36^. Further, *Φ*_r_ increases from ~1.8 × 10^–3^ at an excitation power density of ~6.0 W cm^–2^ to ~5 × 10^–3^ near 30 W cm^–2^ (Fig. 3c). A double logarithmic plot of the respective data (Fig. 3c, inset) reveals two different excitation regimes, one with a slope of 1.8 indicating a biphotonic process, and above 20 W cm^–2^, a regime with a slope of 1.1, signaling a pseudo monophotonic process, presumably due to a high steady concentration of DCB^•–^. Despite a high *I*_th_ value and low *Φ*_r_ values compared to other biphotonic reaction mechanisms^37^, this analysis offers clear evidence for the biphotonic nature and efficiency limits of the catalytic system.

### Pre-association of substrate and photocatalyst

**Fig. 4:**
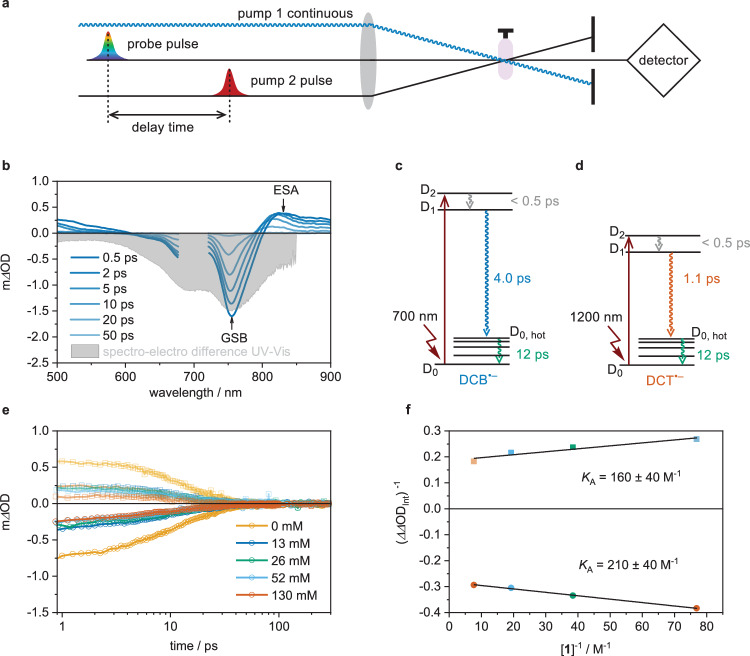
Monitoring pre-association between a radical anion and the substrate. **a** Experimental setup comprised of a 405 nm cw laser (0.5 W), a laser providing pulses of ~190 fs duration at 700 nm (for DCB^•–^) or 1200 nm (for DCT^•–^), and a white-light probe. **b** Transient absorption spectra after various time delays upon excitation of DCB^•–^, generated by continuous 405-nm irradiation of an argon-saturated DMF solution containing 2 mM Ir(ppy)_3_, 10 mM DCB, and 1 M tetra-*n*-butyl ammonium dihydrogen phosphate in deaerated DMF at 20 °C. The gray shaded area is the spectro-electrochemical difference absorption spectrum resulting from the reduction of DCB to DCB^•–^ (Supplementary Fig. 3b) displayed towards the bottom to visualize the expected changes when converting DCB^•–^ to DCB. **c** and **d**, Excited-state decay pathways of ^2*^DCB^•–^ (**c**) and ^2*^DCT^•–^ (**d**) with the associated time constants. D_1_ and D_2_ are the first and the second doublet excited states, D_0,hot_ is a vibrationally non-relaxed ground state. **e** Decay of ^2*^DCB^•–^ monitored by the GSB at 790 nm and the ESA signal at 830 nm in the presence of increasing concentrations of substrate **1**. **f** Benesi-Hildebrand plot assuming a 1:1 pre-association between DCB^•–^ and substrate **1**, based on the integrated differences of changes in optical densities (ΔΔOD_Int_) extracted from the data in **e**.

Two-color pump-pump-probe spectroscopy with sub-picosecond time resolution was used to probe the reactivity of ^2*^DCB^•–^ and ^2*^DCT^•–^. The first pump beam was a 405 nm cw laser (0.5 W) constantly exciting Ir(ppy)_3_ (Fig. 4a), inducing the continuous formation of DCB^•–^ and DCT^•–^. The second pump beam provided pulses of ca. 190 fs duration at 700 nm (to excite DCB^•–^) or 1200 nm (to excite DCT^•–^), and a white light continuum then probed the photoreactions of ^2*^DCB^•–^ and ^2*^DCT^•–^. The resulting transient spectra monitoring the absorption difference caused by the pump pulses for DCB^•–^ (Fig. 4b) and DCT^•–^ (Supplementary Fig. 21) in DMF (without substrate **1**) display excited state absorption (ESA) and ground state bleach (GSB) features. The latter match the spectro-electrochemical difference spectra of the corresponding radical anions, signaling DCB^•–^ and DCT^•–^ disappearance. The overall transient absorption difference data were globally analyzed assuming three successive elementary steps (D_2_ → D_1_ → D_0,hot_ → D_0_) following previous literature (Supplementary Fig. 21)^19^. The obtained lifetimes (*τ*_0_) of 4 ps for ^2*^DCB^•–^ (Fig. 4c) and 1.1 ps for ^2*^DCT^•–^ (Fig. 4d) for the energetically lowest (D_1_) excited states are comparable with the reported D_1_ lifetime of ^2*^DCA^•–^^19^.

If the photoinduced SET step between ^2*^DCB^•–^ and substrate **1** were diffusion-controlled, the maximally achievable quantum yield (*Φ*_r_) for the BHAS reaction would be limited (among other factors) by the efficiency (*η*) of that initial SET step. Based on the D_1_ lifetime of ^2*^DCB^•–^ (4 ps) and the employed concentration of substrate **1** (40 mM) in the BHAS reaction, a maximum SET efficiency of 3.2 × 10^–3^ is estimated for the pseudo monophotonic regime (Supplementary section 6). This *η*-value conflicts with the observed maximum reaction quantum yield of 5 × 10^–3^ (Fig. 3c), even if all elementary reaction steps of the mechanism in Fig. 3a (after initial photoinduced SET) occurred quantitatively. Evidently, pre-association between DCB^•–^ and **1** is needed to facilitate a significantly faster static photoinduced SET step than what is possible based on diffusion control^13,23–26,38^, to account for the observed BHAS reaction quantum yield.

To probe this pre-association between DCB^•–^ and **1**, the two-color pump-pump-probe experiments described above were performed in the presence of increasing concentrations of **1**, similar to a titration experiment (Fig. 4e). No additional spectral bands were detectable because intermediate **I** (the direct photo-reduction product, Fig. 3a) is not expected to have any diagnostic signatures in the observable spectral range, and the photo-oxidation product is DCB, which absorbs in the UV (Fig. 2a). The decay kinetics are essentially unchanged for concentrations of **1** up to 130 mM, because diffusion-controlled bimolecular reaction can only occur with an efficiency of <0.004 under the relevant conditions (Supplementary section 6). However, the amplitude of the transient absorption signal decreases with increasing concentration of **1** while keeping all other parameters constant (Fig. 4e), indicating static excited-state quenching. The decreasing changes in optical density (ΔOD) upon increased concentration of **1** at 790 nm (GSB) and 830 nm (ESA) were quantified by integrating the individual decay traces over time, yielding 5 different ΔOD_Int_ values. The changes of these ΔOD_Int_ values as a function of substrate concentration relative to the ΔOD_Int_ value measured in the absence of substrate is captured by 4 ΔΔOD_Int_ values, reflecting a difference of changes in optical densities. By plotting the reciprocal of these 4 values ((*ΔΔ*OD_Int_)^–1^) against the reciprocal concentration of substrate **1**, a Benesi-Hildebrand plot is obtained (Fig. 4f), in the framework of which the obtained data is compatible with a 1:1 stoichiometry of pre-association between radical anion and substrate. Linear regression yielded an average association constant (*K*_a_) of 185 ± 40 M^–1^. Treating this *K*_a_ value as an equilibrium constant, the relationship Δ*G* = –RT ln(*K*_a_), where R is the universal gas constant and *T* = 293 K^39^, provides a free energy of 13 kJ mol^–1^ for the association between DCB^•–^ and **1** in DMF. Anion – π interactions are often on the order of 20–50 kJ mol^–1^^40^, and a recent study reported a calculated free energy of approximately 21 kJ mol^–1^ for the association between an aminium radical cation and chlorobenzene^13^. It seems that the driving force for radical ion–substrate pre-association is sizeable, and this could form the basis for rapid static SET reactions in more cases than recognized until now.

## Discussion

The sensitized conPET concept established herein mimics the two-chromophore excitation strategy of natural photosynthesis (Fig. 1b, c) and provides access to reduction potentials near –3.2 V versus SCE, directly exploitable for C(sp^2^)―Cl and C(sp^2^)―F bond activation based on pre-associated radical anion – substrate pairs. Mechanistic work until now has indicated that direct radical ion photoreactions are implausible in many cases owing to their picosecond excited-state lifetimes and possible degradation processes, leading to closed-shell compounds that can become the true photoreactive species^16,41^. Our study demonstrates that pre-association between radical ions and suitable substrates can occur with sizeable free energies, approaching those used for template effects in supramolecular chemistry or other forms of (thermal) catalysis^42^. As a photoreaction progresses and the substrate concentration diminishes, pre-association could become less effective, and it seems conceivable that other mechanistic pathways, perhaps including photo-degradation products, could become more important over time. The notion that a photoredox reaction does not necessarily proceed through a single mechanistic pathway seems certainly warranted^43,44^. Nevertheless, improved pre-association by chemical design has the potential to serve as a broadly applicable concept that can enable new photochemistry beyond the diffusion limit. Such research approaches could complement more mainstream strategies geared at elongating the excited-state lifetimes of photocatalysts^45^. The reactions explored herein proceed from the lowest-energetic (D_1_) excited states, but in principle, photo-reactivity from higher excited states might be readily possible in radical ion–substrate aggregates. Synthetic studies provide hints at such anti-Kasha reactivity^13^. The insights gained herein provide a rational basis for fundamentally new photochemistry beyond the current kinetic and thermodynamic limits.

### Supplementary information

Supplementary Information

Peer Review File

## Data Availability

All the data that support the findings of this paper are available via Figshare at 10.6084/m9.figshare.25225673. All data are available from the corresponding author upon request.
