# Supplementary material for: Picosecond reactions of excited radical ion super-reductants
Source: Nat Commun. 2024 Jun 4;15:4738. doi: 10.1038/s41467-024-49006-5 (PMC11150445; doi:10.1038/s41467-024-49006-5)
Supplement: Supplementary file 1 — Supplementary Information [file 41467_2024_49006_MOESM1_ESM.pdf]

Supplementary Information for

# Picosecond reactions of excited radical ion super-reductants

Björn Pfund, Deyanira Gejsnæs-Schaad, Bruno Lazarevski, Oliver S. Wenger*

Department of Chemistry, University of Basel, St. Johanns-Ring 19, 4056 Basel, Switzerland

*Corresponding author: Oliver S. Wenger; e-mail: oliver.wenger@unibas.ch

**Contents**

1. General experimental details ........ 3
2. Synthetic procedures and sample characterization ........ 7
3. Establishing the working principle of the sensitized conPET mechanism ........ 9
4. Photocatalysis of aryl radical substitution reactions via reductive dehalogenations ........ 19
5. Reaction quantum yield ........ 29
6. Ultrafast transient UV-Vis absorption spectroscopy ........ 31
7. NMR spectra ........ 35
8. References ........ 40

## 1. General experimental details

### *Used chemicals*

Synthetic procedures and characterization data for the synthesized compounds are given in Section 2. All synthesized products are literature known. Unless otherwise indicated, solvents and reagents used for optical spectroscopy and irradiation experiments were obtained commercially in high purity and used as received. (*N*,*N*-dimethylformamide, anhydrous, 99.8%, Sigma-Aldrich; $CH_2Cl_2$, anhydrous, ≥99.8%, Sigma-Aldrich; acetonitrile, anhydrous, 99.8%, Sigma-Aldrich; *N*,*N*-diisopropylethylamine, ≥99%, Sigma-Aldrich; *N*,*N*-dimethylaniline, 99%, Sigma-Aldrich; 4,4'-dicyanobiphenyl, 97%, Sigma-Aldrich; *α*,*α*,*α*-trifluorotoluene, anhydrous, ≥99%, Sigma-Aldrich; 2-fluoro-*N*-phenylaniline, 97%, Apollo; 2-chloro-*N*-phenylaniline, 97%, Apollo; 2-bromo-*N*-phenylaniline, ≥98.0%, TCI chemicals; tetra-*n*-butylammonium hexafluorophosphate, 98%, Sigma-Aldrich; tetra-*n*-butylammonium dihydrogen phosphate, 98%, Sigma-Aldrich).

### *Chromatography and basic analytical instrumentation*

Flash chromatography was performed with SiliCycle silica gel 60 (230-400 Mesh). NMR experiments were performed at 298 K on Bruker Avance III or III HD instruments, two or four-channel NMR spectrometers operating at 250.13, 400.13, or 600.13 MHz proton frequency. The NMR spectra were recorded in the indicated deuterated solvent supplied by Cambridge Isotope Laboratories. The NMR spectrometers were equipped with a direct-observe 5 mm BBFO smart probe (250 and 400 MHz) or an indirect-detection 5 mm BBI probe (600 MHz). All probes were equipped with actively shielded *z*-gradients (10 A). Chemical shifts (*δ*) are reported in parts per million (ppm) and referenced to the residual solvent peak.[1] The signal multiplicities are reported in Hz as: s = singlet, d = doublet, t = triplet, q = quartet, and m = multiplet. High-resolution electrospray ionization (HR-ESI) mass spectrometry was recorded by Dr. Michael Pfeffer at the University of Basel on a Bruker MaXis 4G QTOF ESI mass spectrometer. For enhanced ionization, 5 drops of a 0.2 mM $AgNO_3$ solution were added, resulting in the detection of the $[M+Ag]^+$ adducts.

### *Electrochemistry*

Cyclic voltammetry (CV) was measured using a Versastat3-200 potentiostat (Princeton Applied Research) using a saturated calomel electrode (SCE) as a reference, a glassy carbon disk as a working electrode, and a silver wire as a counter electrode. All CV measurements were performed at a potential sweep rate of 50 mV/s in a dry and argon-saturated solvent

containing 0.1 M $TBAPF_6$ (tetra-*n*-butylammonium hexafluorophosphate) as supporting electrolyte and 1 mM analyte.

UV-Vis absorption spectro-electrochemistry was performed using argon-saturated dry solvents in 1 mm optical path length cuvettes. To the sample solution containing 1 mM dicyanoarene (DCX) and supporting electrolyte (0.1 M, $TBAPF_6$), a continuous voltage was applied using Pt mesh (working electrode), Pt wire (counter electrode), and Ag/AgCl (reference electrode). The applied voltage was controlled by a Versastat3-200 potentiostat (Princeton Applied Research). The resulting UV-Vis absorption differences were recorded on a Cary 5000 spectrophotometer from Varian.

***Photocatalysis***

Photocatalysis was performed in a sealed NMR tube (Precision Seal rubber septa cap (5-6 mm), Sigma-Aldrich) using $CD_3CN$ as a solvent. The argon-saturated reaction mixture was irradiated with a 447 nm continuous wave (cw) laser (Roithner Lasertechnik) with precisely adjustable radiative power and a specified output stability fluctuation of less than 1%. The output power was measured with a power meter from Coherent (Field MaxII-TOP Laser Power and Energy Meter). The photocatalysis was performed at different light intensities in a passive water-cooled system. Product formation was monitored on Bruker AVANCE III 250 spectrometer by quantitative $^1H$ NMR spectroscopy using trimethyl(phenyl)silane as an internal standard.

***Reaction quantum yield***

The reaction mixture was subjected to three freeze-pump-thaw cycles and stored in an argon-filled glovebox until the experiment was conducted. For each reaction quantum yield determination, a sample volume of 0.6 ml was transferred to a sealed NMR tube (Precision Seal rubber septa cap (5-6 mm), Sigma-Aldrich). The argon-saturated reaction mixture was irradiated with a 447 nm continuous wave (cw) laser (Roithner Lasertechnik) with precisely adjustable radiative power and a specified output stability fluctuation of less than 1%. The output power was measured with a power meter from Coherent (Field MaxII-TOP Laser Power and Energy Meter). For each applied laser power, the laser spot size at the sample position was individually determined using a laser beam profiler (Newport, BM-USB3-SP932U), giving a laser beam size ranging from 0.0156 $cm^2$ to 0.0169 $cm^2$. The photocatalysis was performed at different excitation power densities in a passive water-cooled system. Product formation was monitored on Bruker AVANCE III 250 spectrometer by quantitative $^1H$ NMR spectroscopy using trimethyl(phenyl)silane as an internal standard.

***Optical spectroscopy***

Steady-state UV-Vis absorption and emission spectra were recorded using a Cary 5000 spectrometer (Varian) and a Fluorolog-3-22 instrument from Horiba Join-Yvon. The solutions used for luminescence spectroscopy were strongly diluted (optical density <0.05 at the excitation wavelength) to avoid filter effects. All luminescence spectra were corrected for the wavelength-dependent detection sensitivity of the spectrometer. The luminescence lifetimes of the DCX compounds were determined on a LifeSpec II spectrometer from Edinburgh Instruments using the time-correlated single photon counting (TCSPC) technique and a picosecond pulsed diode laser.

Transient UV-Vis absorption spectroscopy with nanosecond time resolution was performed on an LP920-KS apparatus from Edinburgh Instruments. Excitation of $Ir(ppy)_3$ at 430 – 450 nm (10 – 14 mJ per pulse) was carried out by a frequency-tripled Nd: YAG pulsed laser (Quantel Brilliant, ca. 10 ns pulse width) equipped with an OPO from Opotek. For direct excitation of the DCX compounds at 355 nm, a frequency-tripled Nd: YAG laser (Quantel Q-smart 450, ca. 10 ns pulse width, pulse energy <120 mJ) was used.

The reactivity of the DCT radical anion ($DCT^{\bullet-}$) was probed using nanosecond two-color pump-pump-probe spectroscopy. The primary 355 nm laser pulses were generated from a frequency-tripled Nd: YAG laser (Quantel Q-smart 450, ca. 10 ns pulse width, pulse energy <100 mJ), forming $DCT^{\bullet-}$. The secondary 532 nm pulses were generatd from a frequency-doubled Nd: YAG laser (Quantel Q-smart 450, ca. 10 ns pulse width, pulse energy <120 mJ), to selectively excite $DCT^{\bullet-}$. Synchronization of the two lasers and the detection system was achieved using a 9520 digital delay pulse generator from Quantum Composers.[2,3] The excitation power densities of both lasers were varied by Q-switch delays and were measured with a pyroelectric detector from Ophir. The beam diameter of 0.6 cm of these 355 nm and 532 nm pulse lasers was enlarged using a beam expander (GBE02-A from Thorlabs) to ensure homogeneous excitation of the whole detection volume (about 1.2 $cm^3$). The lasers were carefully adjusted immediately before starting the measurements. All transient absorption spectra were detected on an iCCD camera from Andor, whereas kinetic traces at a single wavelength were recorded using a photomultiplier tube.

Transient UV-Vis absorption spectra with sub-picosecond time resolution were obtained using a HARPIA-TA spectrometer from Light Conversion in combination with a PHAROS laser (Light Conversion, Yb: KGW laser, source wavelength = 1030 nm, pulse duration = ~190 fs, repetition rate = 50 kHz, output power = 1.0 W, pulse energy = 0.2 mJ). The pump light was generated by an optical parametric amplifier ORPHEUS from Light Conversion, using 90% of the fundamental pulse energy. The probe light was generated by a sapphire white light crystal (5 mm thickness) with 10% energy of the fundamental pulse, resulting in a probe pulse covering

the spectral range from 520 nm to 900 nm. Using a global fitting procedure, the obtained transient absorption UV-Vis spectra were analyzed by CarpetView software from Light Conversion.

A 405 nm cw laser (Roithner Lasertechnik) with an output power of 500 mW and an output stability fluctuation of less than 1% was employed for the pump-pump-probe experiments to investigate the excited state dynamics of $DCX^{\bullet-}$.

## 2. Synthetic procedures and sample characterization

***1-(2-chlorobenzyl)-1H-pyrrole***

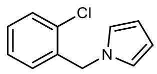


Pyrrole (1.14 mL, 1.11 g, 17 mmol) was added dropwise to a stirred suspension of NaH (60 % in mineral oil, 720 mg, 18 mmol) in dry DMF (30 mL) and stirred for 10 minutes. 2-Chlorobenzylbromide (1.94 mL, 3.08 g, 15mmol) was slowly added, and the mixture was stirred for 14 h at 25 °C, quenched with water, and extracted with ethyl acetate. The combined organic phases were dried over anhydrous $Na_2SO_4$, and the solvent was removed in *vacuo*. The crude product was purified by column chromatography ($SiO_2$, cyclohexane / EtOAc 99:1) to afford 1-(2-chlorobenzyl)-1H-pyrrole (2.53 g, 13 mmol, 88 %) as a colorless liquid. Analytical data match those reported in the literature.[4]

$^1$H NMR (400 MHz, $CDCl_3$): $\delta$ = 7.35 (dd, $J$ = 7.2, 1.8 Hz, 1H,), 7.17 (m, 2H), 6.74 – 6.72 (m, 1H), 6.69 (t, $J$ = 2.1 Hz, 2H), 6.20 (t, J = 2.1 Hz, 2H), 5.14 (s, 2H) ppm.

$^{13}$C NMR (126 MHz, $CDCl_3$): $\delta$ = 136.2, 132.5, 129.4, 128.9, 128.5, 127.3, 121.4, 108.8, 50.0 ppm.

ESI-MS: m/z = $[M+Ag]^+$ calcd. for $C_{11}H_{10}ClNAg$ 297.9553, found 297.9547 $[M+Ag]^+$.

***1-(2-fluorobenzyl)-1H-pyrrole***

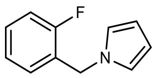


Pyrrole (1.14 mL, 1.11 g, 17 mmol) was added dropwise to a stirred suspension of NaH (60 % in mineral oil, 101 mg, 2.53 mmol, 1.15 eq.) in dry DMF (30 mL) and stirred for 10 minutes. 2-Fluorobenzylbromide (1.81 mL, 2.84 g, 15 mmol) was slowly added, and the mixture was stirred for 14 h at 25 °C, quenched with water, and extracted with ethyl acetate. The combined organic phases were dried over anhydrous $Na_2SO_4$, and the solvent was removed in *vacuo*. The crude product was purified by column chromatography ($SiO_2$, cyclohexane / EtOAc 99:1) to afford 1-(2-fluorobenzyl)-1H-pyrrole (2.35 g, 13 mmol, 89 %) as a colorless liquid. Spectroscopic data match those reported in the literature.[5]

$^1$H NMR (400 MHz, 273 K, $CDCl_3$): $\delta$ = 7.28 – 7.23 (m, 1H), 7.08 – 7.03 (m, 2H), 6.90 (td, $J$ = 7.6, 2.0 Hz, 1H), 6.71 (t, $J$ = 2.2 Hz, 2H), 6.18 (t, $J$ = 2.2 Hz, 2H), 5.11 (s, 2H) ppm.

$^{13}$C NMR (126 MHz, $CDCl_3$): $\delta$ = 161.4 – 159.0 (d, $J$ = 247 Hz), 129.5 (d, $J$ = 8.3 Hz), 129.2 (d, $J$ = 4.2 Hz), 125.5 (d, $J$ = 14.8 Hz), 124.5 (d, $J$ = 4.2 Hz), 121.2, 115.4 (d, $J$ = 21 Hz), 108.7, 47.0 (d, $J$ = 4.7 Hz) ppm.

$^{19}$F NMR (376 MHz, 273 K, $CDCl_3$): $\delta$ = –118.9 ppm.

ESI-MS: m/z = $[M+Ag]^+$ calcd. for $C_{11}H_{10}FNAg$ 281.9848, found 281.9843 $[M+Ag]^+$.

***5H-pyrrolo[2,1-a]isoindole***

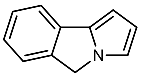


To a solution containing 1-(2-bromobenzyl)-1H-pyrrole (1.18 g, 5 mmol), tricyclohexylphosphonium tetrafluoroborate (0.18g, 0.50 mmol), and $Cs_2CO_3$ (4.89 g, 15 mmol) in dry 1,4-dioxane (20 mL), palladium acetate (0.056 g, 0.25 mmol) was added under nitrogen. The reaction mixture was stirred for 16 hours at 100 °C, quenched with water, and extracted with ethyl acetate. The combined organic phases were dried over anhydrous $Na_2SO_4$, and the solvent was removed in *vacuo*. The crude product was purified by column chromatography ($SiO_2$, cyclohexane / EtOAc 99:1) and recrystallized in methanol to afford 5H-pyrrolo[2,1-a]isoindole (2.35 g, 13 mmol, 89%) as pale pink crystals. Spectroscopic data match those reported in the literature.[6]

$^1$H NMR (400 MHz, $CDCl_3$): $\delta$ = 7.47 (d, $J$ = 7.6 Hz, 1H), 7.38 – 7.28 (m, 2H), 7.14 (td, $J$ = 7.5, 1.1 Hz, 1H), 6.98 – 6.92 (m, 1H), 6.34 (dd, $J$ = 3.6, 2.6 Hz, 1H), 6.28 (dd, $J$ = 3.5, 1.1 Hz, 1H,), 4.93 (s, 2H) ppm.
$^{13}$C NMR (126 MHz, $CDCl_3$): $\delta$ = 140.2, 138.5, 133.5, 128.0, 124.8, 123.0, 118.6, 116.5, 112.7, 98.0, 50.3 ppm.
ESI-MS: m/z = $[M+Ag]^+$ calcd. for $C_{11}H_9NAg$ 261.9786, found 261.9780 $[M+Ag]^+$.

***4,4''-Dicyano-p-terphenyl***

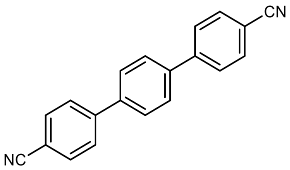


To a mixture containing 4-bromobenzonitrile (5.44 g, 30 mmol) and 1,4-phenylenebisboronic acid (1.65 g, 10 mmol) in argon-saturated dry THF (40 mL) was added 20 mL of a 2 M aqueous solution of $Na_2CO_3$, followed by tetrakis(triphenylphosphine)palladium(0) (400 mg). The reaction mixture was stirred for 12 hours at 85 °C. The resulting white precipitate was filtered, washed with cold THF, and dried under reduced pressure to afford 4,4''-dicyano-*p*-terphenyl as a white solid (2.41 g, 8.6 mmol, 84%), $^1$H, and $^{13}$C NMR spectra are in agreement with the previously reported spectral data.[7] The $^{13}$C NMR experiments were recorded on an ADVANCE III 600 spectrometer using an indirect BBI probe head, whereas the $^1$H NMR experiment was recorded on a Bruker AVANCE III 400 spectrometer.

$^1$H NMR (400 MHz, $CDCl_3$): $\delta$ = 7.71 – 7.65 (m, 12 H) ppm.
$^{13}$C NMR (151 MHz, $CDCl_3$): $\delta$ =144.7, 159.4, 132.8, 128.0, 127.7, 118.8, 111.4 ppm.

## 3. Establishing the working principle of the sensitized conPET mechanism

### Determination of the excited state redox potentials of $DCT^{•–}$ and $DCB^{•–}$

The optical-spectroscopic and electrochemical properties of the DCT and DCB photocatalysts were analyzed to estimate the excited state redox potentials of $DCT^{•–}$ and $DCB^{•–}$. The steady-state optical absorption properties of the supposed DCT and DCB photocatalysts in their neutral and radical mono-anionic forms were investigated (Fig. 2a, main manuscript). DCT and DCB solely absorb below 350 nm with absorption maxima at 307 nm (DCT) and 280 nm (DCB) with similar molar absorption coefficients of 29'200 $M^{–1}$ $cm^{–1}$ for DCT and 27'600 $M^{–1}$ $cm^{–1}$ for DCB (Fig. 2a, main manuscript, dashed traces). Both dicyanoarene derivatives show a fluorescence band with a maximum at 368 nm for DCT and 335 nm for DCB (Fig. 2a, main manuscript, dotted traces) with a singlet excited state ($S_1$) lifetime of 0.86 ns for DCT and 1.19 ns for DCB (Supplementary Fig. 1) in aerated DMF at 20 °C. By analyzing the intersections of absorption and emission spectra (Fig. 2a, main manuscript), the $S_1$ excited state energies ($E_{S1}$) of ~3.7 eV (DCT) and ~4.0 eV (DCB) were determined (Supplementary Table 1).

Cyclic voltammetry measurements in DMF (Supplementary Fig. 2) yielded potentials for one-electron reduction of DCT and DCB of –1.7 V versus SCE and –1.6 V versus SCE, respectively (Supplementary Table 1). Furthermore, a second reversible reduction wave was observed for DCT at –1.9 V versus SCE and for DCB at –2.0 V versus SCE. Spectro-electrochemical UV-Vis difference spectra (Fig. 2a, main manuscript, solid traces) recorded at a potential of −1.5 V versus an Ag pseudo reference electrode show the absorption features of the dicyanoarene radical anions ($DCX^{•–}$) with absorption bands at 1175 nm and 530 nm for $DCT^{•–}$ and at 757 nm and 454 nm for $DCB^{•–}$, in good agreement with previously published data.[8] As no emission from $DCX^{•–}$ can be detected, the lowest doublet excited state ($D_1$) energies ($E_D$) were estimated by taking the point on the low-energy side of the absorption spectra of $DCT^{•–}$ and $DCB^{•–}$, at which 10% of the maximum absorption is reached,[9] resulting in an estimated $E_D$ of 1.0 eV for $^{2*}DCT^{•–}$ and 1.6 eV for $^{2*}DCB^{•–}$ (Supplementary Table 1). The redox potentials ($^{2*}E_{red}$) of the $D_1$ excited $^{2*}DCT^{•–}$ and $^{2*}DCB^{•–}$ were estimated based on the Rehm-Weller equation:[9-11] $^{2*}E_{red}(DCX/^{2*}DCX^{•–}) \approx E_{red}(DCX/DCX^{•–}) – E_D \times e$ with $e$ the elementary charge, the ground state reduction potential ($E_{red}$) of DCT (–1.7 V versus SCE), or DCB (–1.6 V versus SCE) and the estimated $D_1$ energy ($E_D$) of 1.0 eV ($DCT^{•–}$), or 1.6 eV ($DCB^{•–}$). The estimated $D_1$ excited state potentials (Supplementary Table 1) indicate exceptionally strong reducing powers of approximately –2.7 V versus SCE for $^{2*}DCT^{•–}$ and –3.2 V versus SCE for $^{2*}DCB^{•–}$.

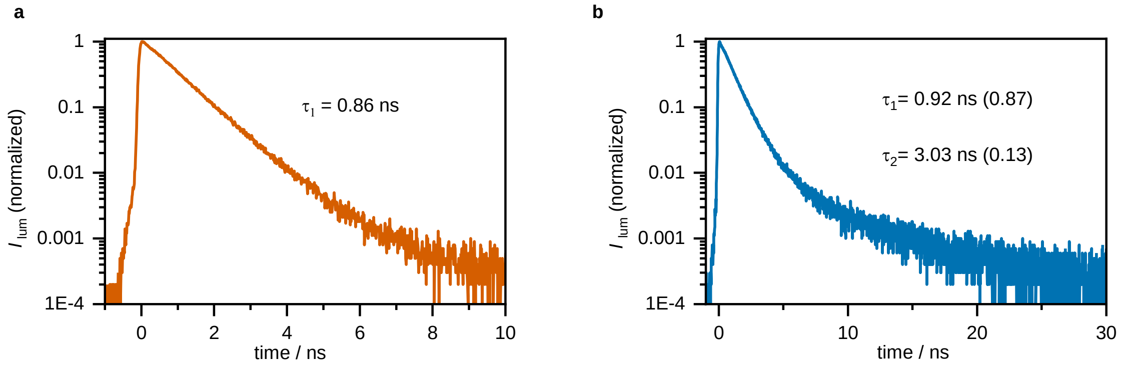


**Supplementary Fig. 1.** Upon pulsed excitation at 375 nm, the singlet excited state ($S_1$) lifetimes of DCT (**a,** c = 1 × $10^{-4}$ M) and DCB (**b,** c = 1 × $10^{-3}$ M) in aerated DMF solution at 20 °C were measured at 390 nm using the TCSPC technique. The bi-exponential excited state decay of $^{1*}$DCB (**b**) is tentatively attributed to self-quenching or excimer formation due to the high concentration used in this experiment, needed because of the weak absorption of DCB at the available excitation wavelength. The lifetime reported in the text for DCB is an amplitude weighted average from a bi-exponential fit, resulting in 1.19 ns (= (0.92 ns × 0.87) + (3.03 ns × 0.13).

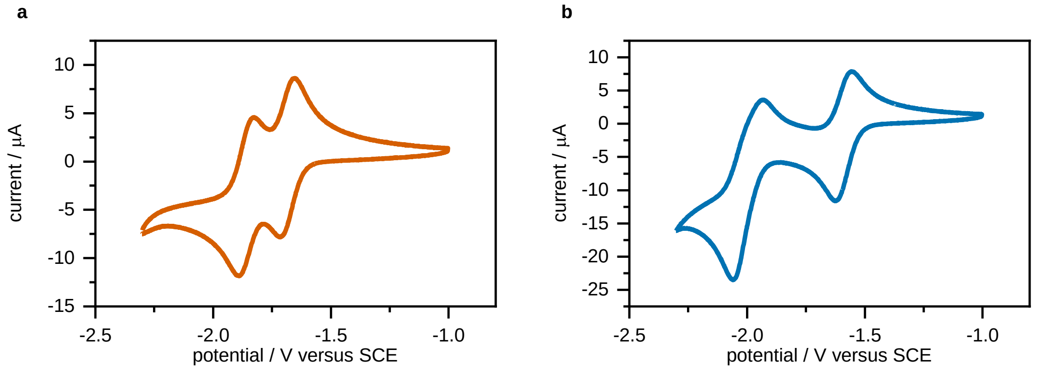


**Supplementary Fig. 2.** Cyclic voltammograms of argon-saturated DMF solutions containing 1 mM DCT (**a**) or 1 mM DCB (**b**), using a saturated calomel reference electrode (SCE). $TBAPF_6$ (0.1 M) was used as a supporting electrolyte, a glassy carbon disk was employed as a working electrode, and a silver wire served as the counter electrode. The potential scan rate was 0.05 V $s^{-1}$.

**Supplementary Table 1.** Summary of selected properties of the investigated DCB and DCT compounds.

| PS | $E_S$[a] / eV | $E_{red}$ [b] ($DCX/DCX^{•–}$) / V versus SCE | $E_{red}$ [b] ($DCX^{•–}/DCX^{2–}$) / V versus SCE | $k_q$[c] / $10^8$ $M^{–1}$ $s^{–1}$ | $E_D$[d] / eV | $^*E_{red}$ [e] ($^{2*}DCX^{•–}/DCX$) / V versus SCE |
|---|---|---|---|---|---|---|
| DCT | 3.6 | –1.7 | –1.9 | 1.7 | 1.0 | –2.7 |
| DCB | 4.0 | –1.6 | –2.0 | 19 | 1.6 | –3.2 |

[a] Singlet excited state energies ($E_S$) in DMF were determined at the intersection between the absorption and fluorescence spectra (Fig. 2a, main manuscript).

[b] Ground state reduction potential in DMF (Supplementary Fig. 2).

[c] Reaction rate constant between $^{3*}Ir(ppy)_3$ and DCT, or between $^{3*}Ir(ppy)_3$ and DCB in DMF (Supplementary Figs. 8 and 9).

[d] Doublet excited state energies ($E_D$) were estimated by taking the point on the low-energy side of the absorption spectra of $DCT^{•–}$ and $DCB^{•–}$, at which 10% of the maximum absorption is reached (Fig. 2a, main manuscript).

[e] The excited state redox potentials of $^{2*}DCX^{•–}$ ($^*E_{red}(^{2*}DCX^{•–}/DCX)$) were calculated using $^*E_{red}(^{2*}DCX^{•–}/DCX) \approx E_{red}(DCX^{•–}/DCX) – E_D \times e$, with e the elementary charge, the ground state potentials ($E_{red}$ ($DCX^{•–}/DCX$), column 3), and the $D_1$ energy ($E_D$, column 6).

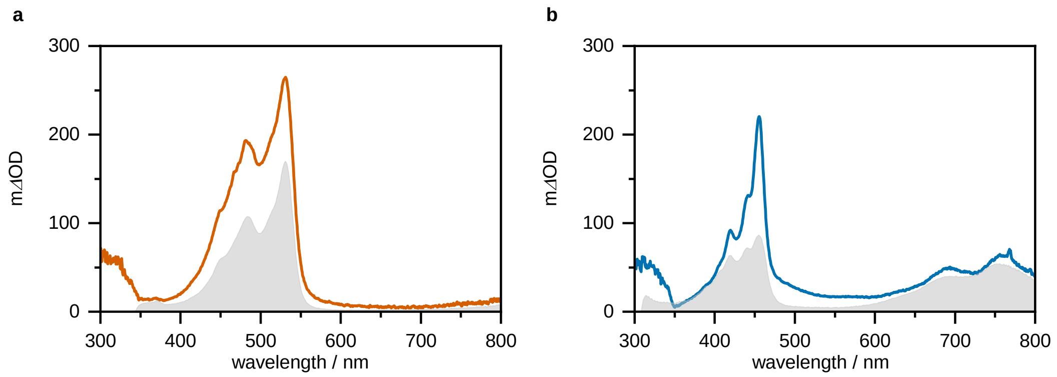


**Supplementary Fig. 3.** Transient UV-Vis absorption spectra of argon-saturated DMF solutions at 20 °C containing 200 mM *N*,*N*-dimethylaniline (DMA) with 2 mM DCT (**a**, orange trace), or 2 mM DCB (**b**, blue trace) after pulsed excitation at 355 nm (25 mJ per pulse), recorded with a time delay of 100 ns and an integration time of 200 ns. The grey areas mark the UV-Vis changes on electrochemical reduction at −1.5 V versus an Ag pseudo reference electrode of argon-saturated DMF solutions containing $TBAPF_6$ (0.1 M) as a supporting electrolyte and 600 µM DCT (**a**) or 600 µM DCB (**b**).

## Nanosecond two-color pump-pump-probe laser flash photolysis

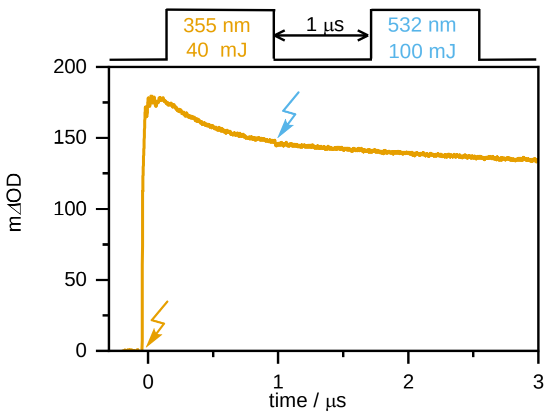


**Supplementary Fig. 4.** Transient UV-Vis absorption kinetic decay of $DCT^{\bullet-}$ monitored at the probe wavelength of 500 nm in a two-color pump-pump-probe experiment (pulse scheme is shown above). The experiment was performed using an argon-saturated DMF solution containing 2 mM DCT and 200 mM DMA, without adding $CH_2Cl_2$. The orange flash indicates the first laser pulse (355 nm, 40 mJ), whereas the blue flash marks the time point (after a delay of 1 µs following the first excitation pulse), at which the second laser pulse (532 nm, 80 mJ) occurred.

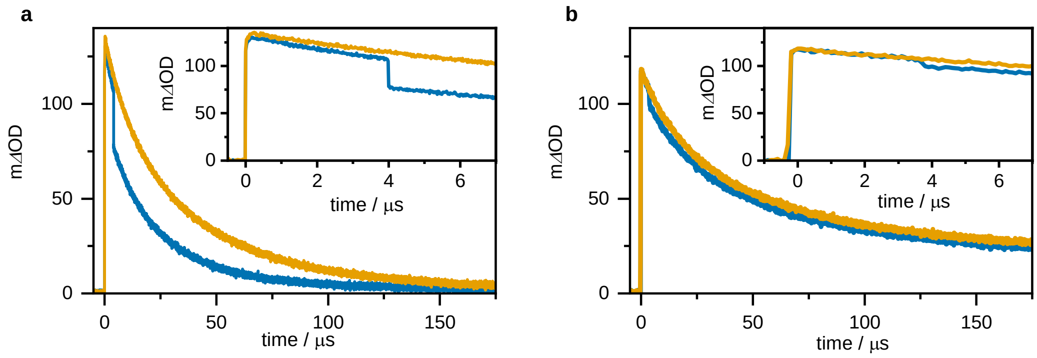


**Supplementary Fig. 5.** Transient UV-Vis absorption kinetic data for $DCT^{\bullet-}$ monitored at 500 nm in a two-color pump-pump-probe experiment. In this experiment, a first 355 nm (40 mJ) laser pulse generated $DCT^{\bullet-}$ (orange traces). After a time delay of 4 µs, a second laser pulse with a wavelength of 532 nm (100 mJ) excited $DCT^{\bullet-}$ (blue traces). The two-color pump-pump-probe experiments were carried out using an argon-saturated DMF solution containing 2 mM DCT, 200 mM DMA, and 280 mM $CH_2Cl_2$ (**a**) or 150 mM *α,α,α*-trifluorotoluene (**b**).

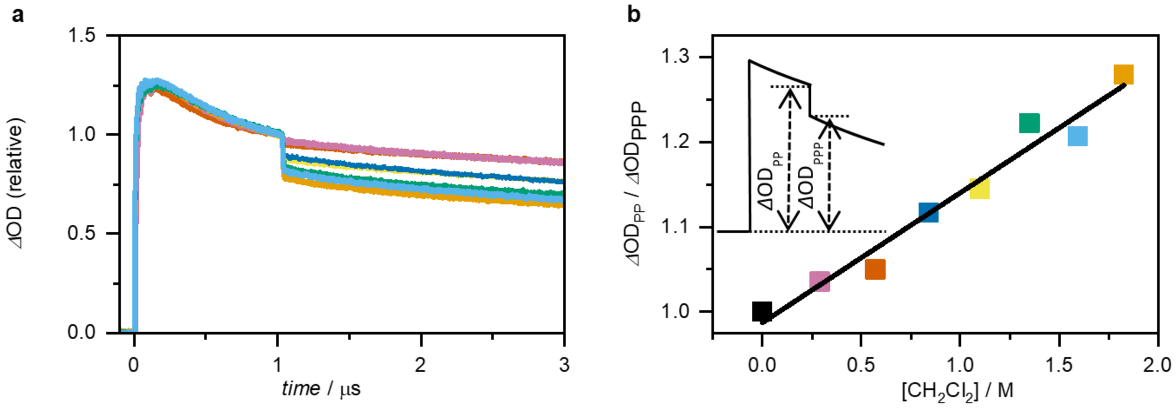


Supplementary Fig. 6. **a**, Transient UV-Vis absorption kinetic decays of $DCT^{•–}$ monitored at 500 nm in a two-color pump-pump-probe experiment. In this experiment, a first 355 nm laser pulse (40 mJ) generated $DCT^{•–}$, followed by a second 532 nm laser pulse (100 mJ) after a time delay of 1 μs, to excite $DCT^{•–}$. These two-color pump-pump-probe experiments were performed using argon-saturated DMF solutions containing 2 mM DCT, 200 mM DMA, and between 0 mM and 1900 mM $CH_2Cl_2$. **b**, Stern-Volmer-like plot (identical to Fig. 2c, main manuscript) based on the two-pulse experiment as a function of increasing $CH_2Cl_2$ concentration. Inset: schematic illustration of the two observables $\Delta OD_{PP}$ (PP = pump-probe; change in optical density at $t$ = 1 μs, recorded immediately before the second pump pulse) and $\Delta OD_{PPP}$ (PPP = pump-pump-probe, change in optical density at $t$ = 1 μs, recorded immediately after the second pump pulse). All decay traces were normalized to a $\Delta OD_{PP}$ value of 1.0. With the resulting Stern-Volmer constant of 0.012 $M^{-1}$ and the $^{2*}DCT^{•–}$ excited state lifetime of 1.1 ps (Fig. 4d), an SET reaction rate constant from $^{2*}DCT^{•–}$ to $CH_2Cl_2$ of 1.1 × $10^{10}$ $M^{-1}$ $s^{-1}$ was determined.

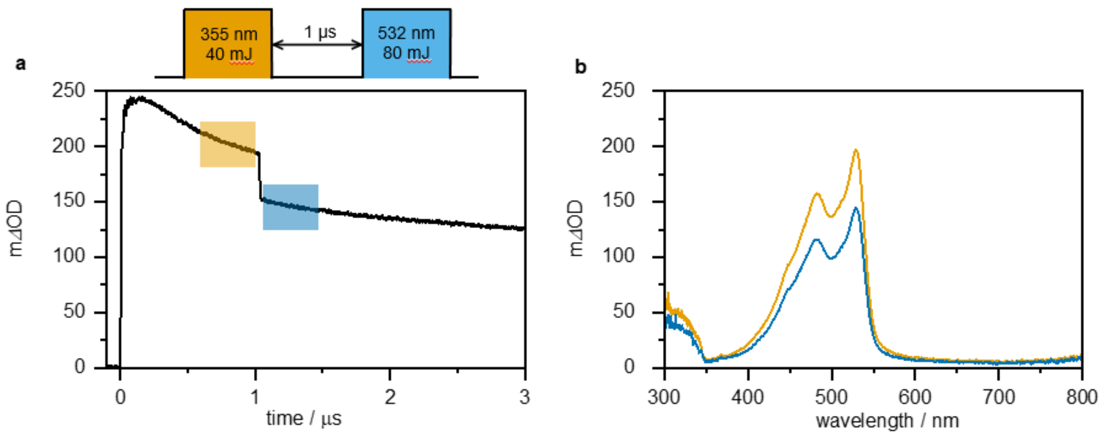


**Supplementary Fig. 7. a**, Transient UV-Vis absorption kinetic decay of $DCT^{\bullet-}$ monitored at 500 nm in a two-color pump-pump-probe experiment (pulse scheme is shown above). In this experiment, a first 355 nm (40 mJ) laser pulse generated $DCT^{\bullet-}$. After a time delay of 1 µs, a second 532 nm (100mJ) laser pulse excited $DCT^{\bullet-}$. This two-color pump-pump-probe experiment was performed using an argon-saturated DMF solution containing 2 mM DCT, 200 mM DMA, and 1900 mM $CH_2Cl_2$. **b**, Transient UV-Vis absorption spectra recorded in the two-time intervals indicated by the orange and blue marked areas in **a**.

## The search for a suitable photosensitizer

To enable the formation of $DCX^{•-}$ with visible light, $Ir(ppy)_3$ (Hppy, 2-phenylpyridine) with its triplet excited state energy of 2.5 eV[12] and an excited state reduction potential of –1.7 V versus SCE[12] was identified as a suitable photosensitizer to directly reduce both DCX compounds. This excited state reduction potential seemed sufficient for reducing DCT and DCB, with their ground state one-electron reduction potentials of –1.7 V and –1.6 V versus SCE (Supplementary Table 1). To determine the reaction rate constant between the triplet excited $Ir(ppy)_3$ and the two DCX compounds, time-resolved luminescence quenching experiments were performed with DCT (Supplementary Fig. 8) and DCB (Supplementary Fig. 9). The resulting bimolecular reaction rate constant ($k_q$) of $1.9 \times 10^9$ $M^{-1}$ $s^{-1}$ for DCB is ten-fold higher than for DCT ($1.7 \times 10^8$ $M^{-1}$ $s^{-1}$), due to the less negative reduction potential of DCB (–1.6 V versus SCE) compared to DCT (–1.7 V versus SCE). However, the high triplet excited state energy of $Ir(ppy)_3$ (2.5 eV) can in principle also lead to a triplet-triplet energy transfer (TTET) reaction. Consequently, transient UV-Vis absorption spectroscopy was employed to identify the possible TTET or SET photoproducts (Supplementary Figs. 8 & 9). The transient UV-Vis absorption spectra of $Ir(ppy)_3$ in the presence of DCB (Supplementary Fig. 8) resulted in strong TA signals at 454 nm and 778 nm, identical to the spectro-electro difference UV-Vis bands in Fig. 2. This strongly suggests the direct formation of $DCB^{•-}$ via photoinduced SET. When exciting $Ir(ppy)_3$ in the presence of DCT, an ESA signal at 500 nm was observed on short time scales (<10 μs). At longer time delays (>50 μs), two distinct TA signals at 490 nm and 532 nm were observed, matching with the spectro-electrochemically generated UV-Vis spectrum of $DCT^{•-}$, indicating partial photoinduced SET. To identify the species causing the additional TA signals at time delays below 10 μs, $[Ir(s^{dF}ppy)_3]^{3-}$ ($s^{dF}$Hppy, 2,6-difluoro-3-(pyridine-2-yl)benzenesulfonate) was used as a photosensitizer. $[Ir(s^{dF}ppy)_3]^{3-}$ has a higher triplet excited state energy of 2.8 eV compared to the previously used $Ir(ppy)_3$ photosensitizer (2.5 eV), but a similar excited state reduction potential,[13-15] making TTET process more competitive with photoinduced SET. The obtained TA spectra with $[Ir(s^{dF}ppy)_3]^{3-}$ (Supplementary Fig. 9d) exhibit only a TA signal at 500 nm (instead of the main band near 520 nm attributable to the SET photo-product), similar to what has been observed with $Ir(ppy)_3$ at short delay times (<10 μs). This confirms that there are competing TTET and photoinduced SET reactions, but in the presence of an excess of an electron donor such as DMA, only transient absorption signals associated with $DCT^{•-}$ are observed, indicating SET from the electron donor to $^{3*}$DCT.[9]

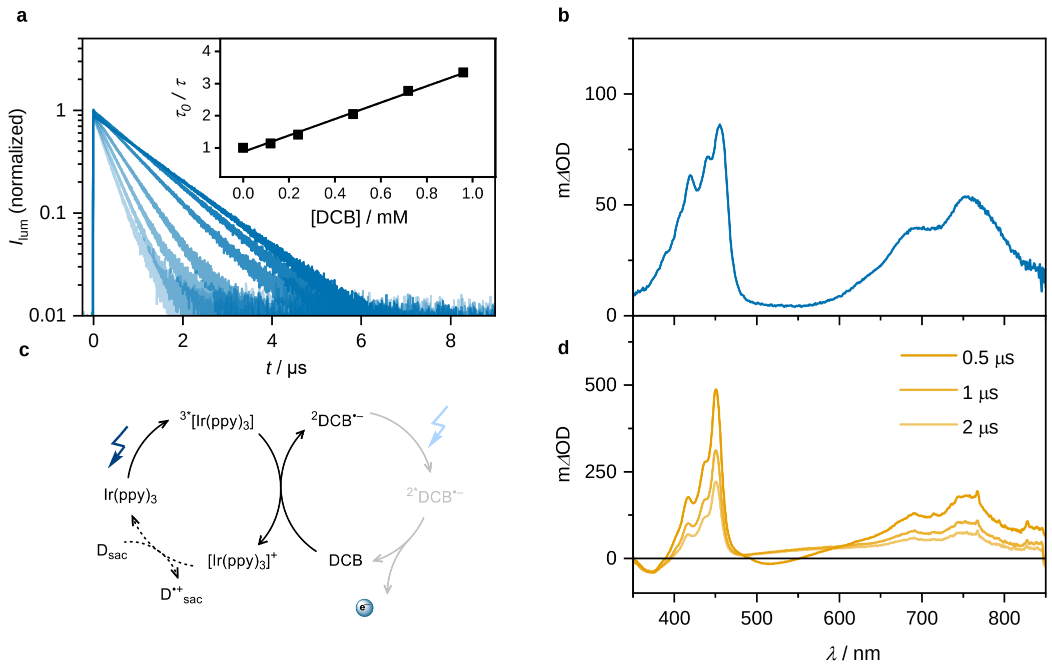


**Supplementary Fig. 8. a**, Emission decay traces at 525 nm of Ir(ppy)$_3$ ($c$ = 50 µM) in an argon-saturated $CH_3CN$ solution in the absence and presence of different DCB concentrations after 450 nm pulsed excitation (13 mJ per pulse). Inset, obtained Stern-Volmer plot, from which a bimolecular reaction rate constant of $1.9 \times 10^9$ $M^{-1}$ $s^{-1}$ was extracted. **b**, Spectro-electrochemical UV-Vis difference spectrum of an argon-saturated DMF solution containing 600 µM DCB and 0.1 M tetra-*n*-butylammonium hexafluorophosphate ($TBAPF_6$), at an applied potential of −1.5 V versus an Ag pseudo reference electrode (blue solid trace). **c**, Simplified mechanistic representation of the sensitized ConPET mechanism with an initial photoinduced SET elementary step from $^{3*}$Ir(ppy)$_3$ to DCB. The grey arrows indicate the second photon absorption cycle, and the dashed arrows indicate the regeneration of the initial form of the iridium(III) complex, closing the first photon absorption cycle. **d**, Transient UV-Vis absorption spectra upon 428 nm pulsed excitation (13 mJ per pulse) of an argon-saturated DMF solution containing Ir(ppy)$_3$ (50 µM) and DCB (10 mM) after different time delays.

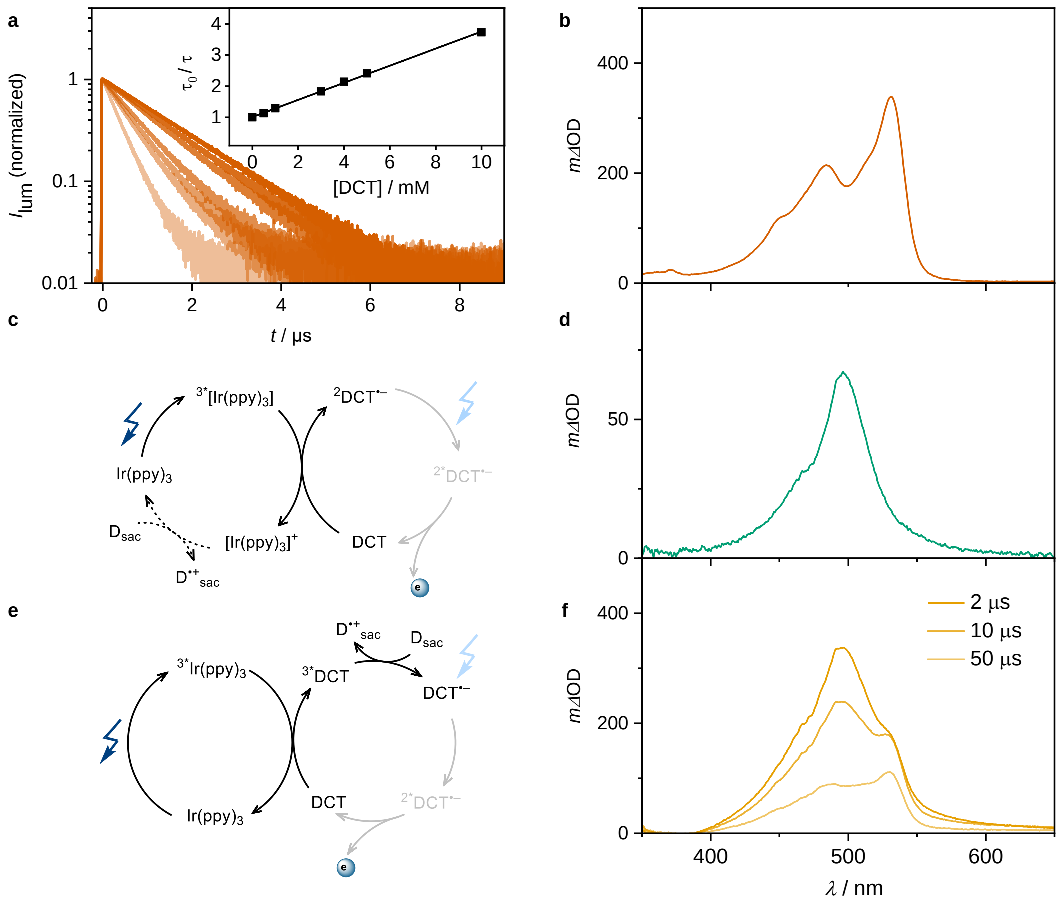


**Supplementary Fig. 9. a**, Emission decay traces at 525 nm of Ir(ppy)$_3$ ($c$ = 50 µM) in an argon-saturated $CH_3CN$ solution in the absence of DCT and presence of different DCT concentrations after pulsed excitation at 450 nm (13 mJ per pulse). Inset, obtained Stern-Volmer plot, from which a bimolecular reaction rate constant of 1.7 × $10^8$ $M^{-1}$ $s^{-1}$ was extracted. **b**, Spectro-electrochemical UV-Vis difference spectrum of $DCT^{\bullet-}$ in an argon-saturated DMF solution. The spectrum was recorded using a solution containing 1 mM DCT and 0.1 M tetra-*n*-butylammonium hexafluorophosphate ($TBAPF_6$), at an applied potential of −1.5 V versus an Ag pseudo reference electrode, leading to one-electron reduction of DCT. **c**, Simplified mechanistic representation of the sensitized ConPET mechanism with an initial SET process from $^{3*}$Ir(ppy)$_3$ to DCT. The grey arrows indicate the second photon absorption cycle, whereas the dashed arrows indicate the regeneration of the initial form of the iridium(III) complex, closing the first photon absorption cycle. **d**, Triplet excited DCT ($^3$*DCT) in $CH_3CN$, generated by a sensitized triplet-triplet energy transfer reaction between [Ir(s$^{dF}$ppy)$_3$]$^{3-}$ (s$^{dF}$Hppy = 2,6-difluoro-3-(pyridine-2-yl)benzensulfonate) with a triplet excited state energy of 2.81 eV[13-15] and DCT. The spectrum was recorded after a time delay of 1 µs following pulsed excitation at 440 nm (15 mJ per pulse) of an argon-saturated DMF solution containing 200 µM [Ir(s$^{dF}$ppy)$_3$]$^{3-}$ and

5 mM DCT. **e**, Simplified mechanistic representation of the sensitized ConPET mechanism with an initial TTET process forming $^{3*}$DCT, followed by a SET from a sacrificial electron donor ($D_{sac}$). The grey arrows indicate the second photon absorption cycle, and the dashed arrows indicate the regeneration of the initial form of the iridium(III) complex, closing the first photon absorption. **f**, Transient UV-Vis absorption spectra upon pulsed excitation at 428 nm (13 mJ per pulse) of an argon-saturated DMF solution containing $Ir(ppy)_3$ (50 µM) and DCT (10 mM) recorded after different time delays.

## 4. Photocatalysis of aryl radical substitution reactions via reductive dehalogenations

### *Photo-degradation of DCB in the presence of DIPEA*

We tested the photostability of DCB in the presence of an electron donor, such as *N*,*N*-diisopropylethylamine (DIPEA), to probe whether any photoactive decomposition products form. For this purpose, a solution containing Ir(ppy)$_3$ (2 mM), DCB (20 mM), and DIPEA (200 mM) in argon-saturated $CD_3CN$ was irradiated for 21 hours with a 447 nm cw laser (1 W) and subsequently analyzed using $^1$H NMR spectroscopy. After 21 hours of irradiation, the $^1$H NMR spectrum showed no remaining signal associated with DCB. The decomposition product was identified via $^1$H NMR spectroscopy as 4'-ethyl-[1,1'-biphenyl]-4-carbonitrile (Supplementary Fig. 10), matching the literature known $^1$H NMR spectrum of this compound.[16]

$^1$H NMR (400 MHz, $CDCl_3$): $\delta$ = 7.72 – 7.66 (m, 4H), 7.53 – 7.51 (m, 2H), 7.32– 7.31 (m, 2H), 2.71 (q, $J$ = 7.6 Hz, 2H), 1.28 (t, $J$ = 7.6 Hz, 3H) ppm.

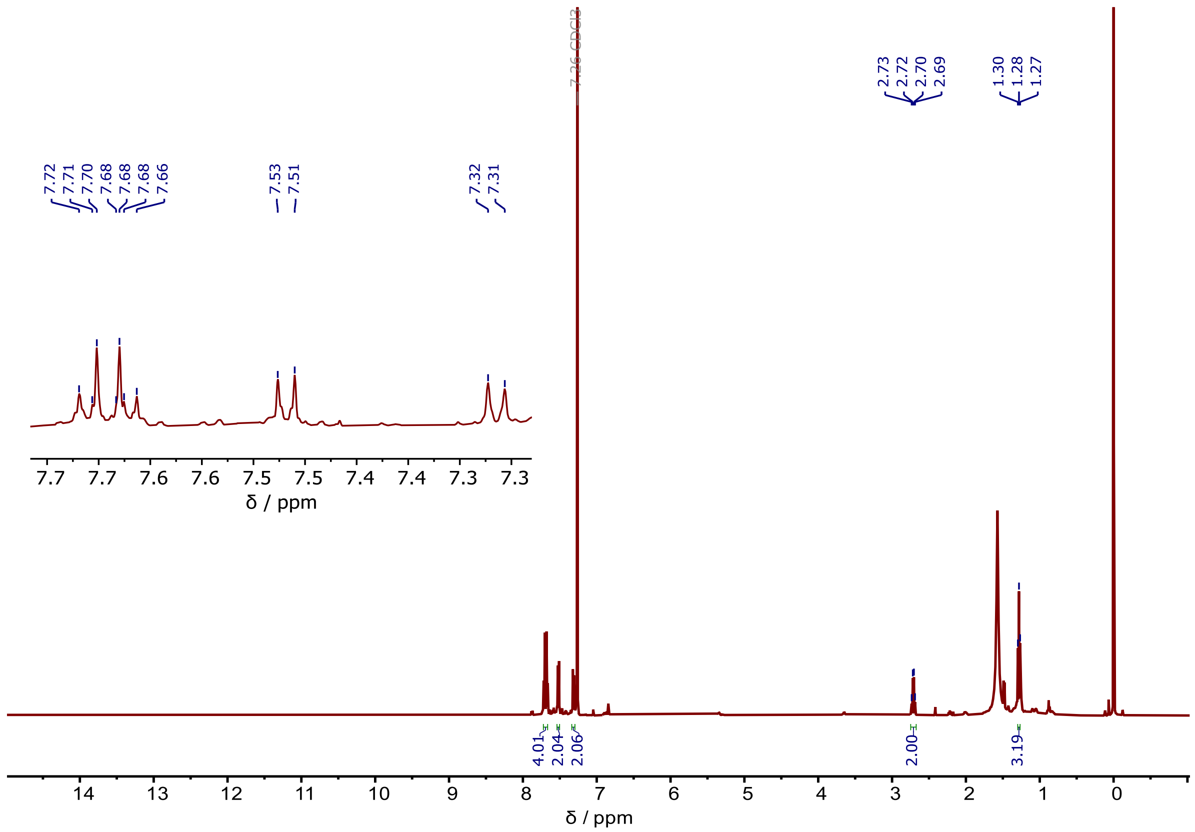


**Supplementary Fig. 10.** $^1$H NMR spectrum recorded after 21 hours of 447 nm irradiation (1 W) of a solution containing Ir(ppy)$_3$ (2 mM), DCB (20 mM), and DIPEA (200 mM) in argon-saturated $CD_3CN$, matching the literature known spectrum of 4'-ethyl-[1,1'-biphenyl]-4-carbonitrile.[16]

***Base-promoted homolytic aromatic substitution (BHAS)***

The reaction mixture containing 40 mM substrate **1**, 800 μM Ir(ppy)$_3$, 4 mM DCB, and 80 mM TMP (2,2,6,6-tetramethylpiperidine) in deaerated $CD_3CN$ was irradiated for 21 hours with a 447 nm continuous wave laser with a maximum output energy of 2 W. The reaction progress was monitored by following the benzylic $^1H$ NMR resonances of substrate **1** at 5.2 ppm (Supplementary Fig. 11, marked in red) and product **3** at 4.9 ppm (Supplementary Fig. 11, marked in blue) in $CD_3CN$ using trimethyl(phenyl)silane as an internal standard. The hydrochlorination side product (1-benzylpyrrole) was identified with a yield of ~5% (Supplementary Fig. 11, marked in orange). Several control experiments were conducted (Supplementary Fig. 13), and their outcomes are summarized in Supplementary Table 2 (entries 1-5).

Given that the aryl-halide bond activation is expected to be the rate-determining step for the investigated BHAS and other intra-molecular nucleophilic radical substitution reactions, several alternative photosensitizers are in principle suitable for the sensitized ConPET mechanism, provided they can supply sufficient excited state reduction power of at least –1.6 V versus SCE, in order to generate $DCB^{\bullet-}$ photochemically. Consequently, we explored various classical iridium-based and organic photosensitizers with excited-state reduction potentials below –1.6 V versus SCE to initiate the first SET in order to generate $DCB^{\bullet-}$ for the BHAS reaction of substrate **1** (Supplementary Table 2). All tested iridium-based[13-15] and organic photosensitizers[17,18] (capable of forming $DCB^{\bullet-}$) resulted in comparable product formation, demonstrating the versatility of our newly developed sensitized conPET strategy. Evidently, there is no strict need for precious metal-based photosensitizers.

**Supplementary Table 2.** Optimization of the intramolecular-based promoted aromatic substitution (BHAS) reaction of 1-(2-chlorobenzyl)-1H-pyrrole (substrate **1**) to product **3**.

| Entry | Variation of standard conditions [a] | $^1$H NMR-yield (conversion) |
|---|---|---|
| 1 | - | 62% (72%) |
| 2 | without TMP | 0% (0%) |
| 3 | without DCB | 0% (0%) |
| 4 | without $Ir(ppy)_3$ | 0% (0%) |
| 5 | without irradiation | 0% (0%) |
| 6 | adding tetra-*n*-butylammonium dihydrogen phosphate (1M) [b] | 29% (44%)[g] |
| 7 | using $[Ir(sppy)_3]^{3-}$ [c] | 15% (33%)[g] |
| 8 | using $Ir(Fppy)_3$ [d] | 31% (42%)[g] |
| 9 | using 3DPA2FBN [e] | 20% (27%) |
| 10 | using Phenox O-PC [f] | 17% (21%) |

[a] Standard conditions: 40 mM substrate, 800 µM $Ir(ppy)_3$, 4 mM DCB, and 80 mM 2,2,6,6-tetramethylpiperidine (TMP) in $CD_3CN$. (Concentrations of other photosensitizers were identical, where applicable).

[b] As bulky organic salts might be able to stabilize radical anions, tetra-*n*-butylammonium dihydrogen phosphate was added in an attempt to improve the overall catalytic performance.[19,20]

[c] $[Ir(sppy)_3]^{3-}$ (sHppy = 3-(pyridin-2-yl)benzenesulfonate).

[d] $Ir(Fppy)_3$ (FHppy = 2-(4-fluorophenyl)pyridine).

[e] 3DPA2FBN = 2,4,6-tris(diphenylamino)-3,5-difluorobenzonitrile.

[f] Phenox O-PC = 3,7-di(4-biphenyl) 1-naphthalene-10-phenoxazine.

[g] Decomposition of DCB (< 50 %) over the irradiation time of 21 hours.

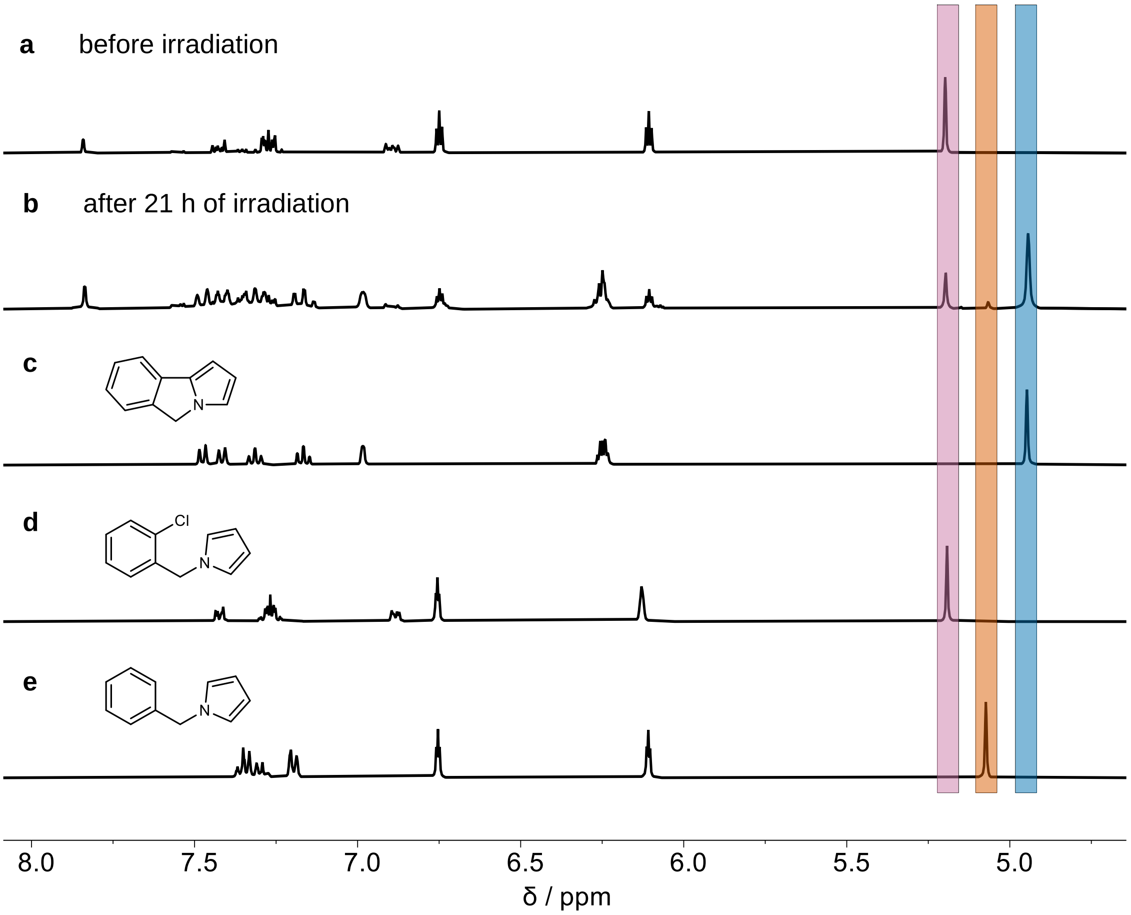


**Supplementary Fig. 11.** $^1$H NMR spectra monitoring the intramolecular base-promoted homolytic aromatic substitution (BHAS) reaction of 1-(2-chlorobenzyl)-1H-pyrrole **1** (red background) to 5H-pyrrolo[2,1-a]isoindole **3** (blue background) and the hydrodehalogenation side product 1-benzylpyrrole (orange background). The signal at 7.8 ppm originates from DCB. **a** & **b**, $^1$H NMR spectra of the reaction mixture containing 800 µM Ir(ppy)$_3$, 4 mM DCB, 80 mM TMA, and 40 mM 1-(2-chlorobenzyl)-1H-pyrrole (**1**) in argon-saturated $CD_3CN$ before (**a**) and after 21 hours of 447 nm cw laser (2 W) irradiation (**b**). **c** – **e**, reference spectra of the desired product 5H-pyrrolo[2,1-a]isoindole **3** (**c**), starting material 1-(2-chlorobenzyl)-1H-pyrrole **1** (**d**), and the hydrodehalogenation side product 1-benzylpyrrole (**e**).

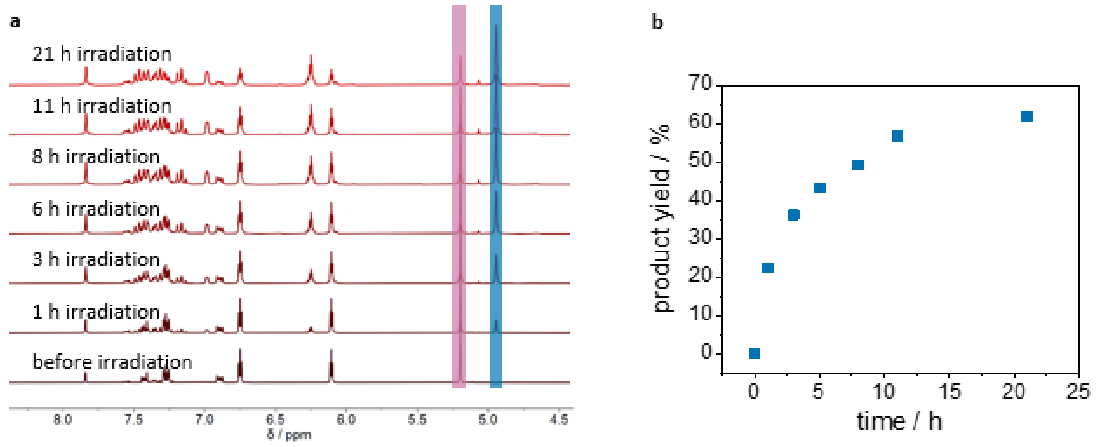


**Supplementary Fig. 12. a**, $^{1}H$ NMR spectra monitoring the base-promoted homolytic aromatic substitution (BHAS) reaction of 40 mM 1-(2-chlorobenzyl)-1H-pyrrole **1** (red background) to 5H-pyrrolo[2,1-a]isoindole **3** (blue background) using Ir(ppy)$_3$ (800 μM), DCB (4 mM), TMP (80 mM) in argon-saturated $CD_3CN$ at different irradiation times with a 447 nm cw laser (2 W). **b**, Product yield of the BHAS reaction of 1-(2-chlorobenzyl)-1H-pyrrole **1** to 5H-pyrrolo[2,1-a]isoindole **3** after different irradiation times. Product yields were determined based on trimethyl(phenyl)silane as an internal standard.

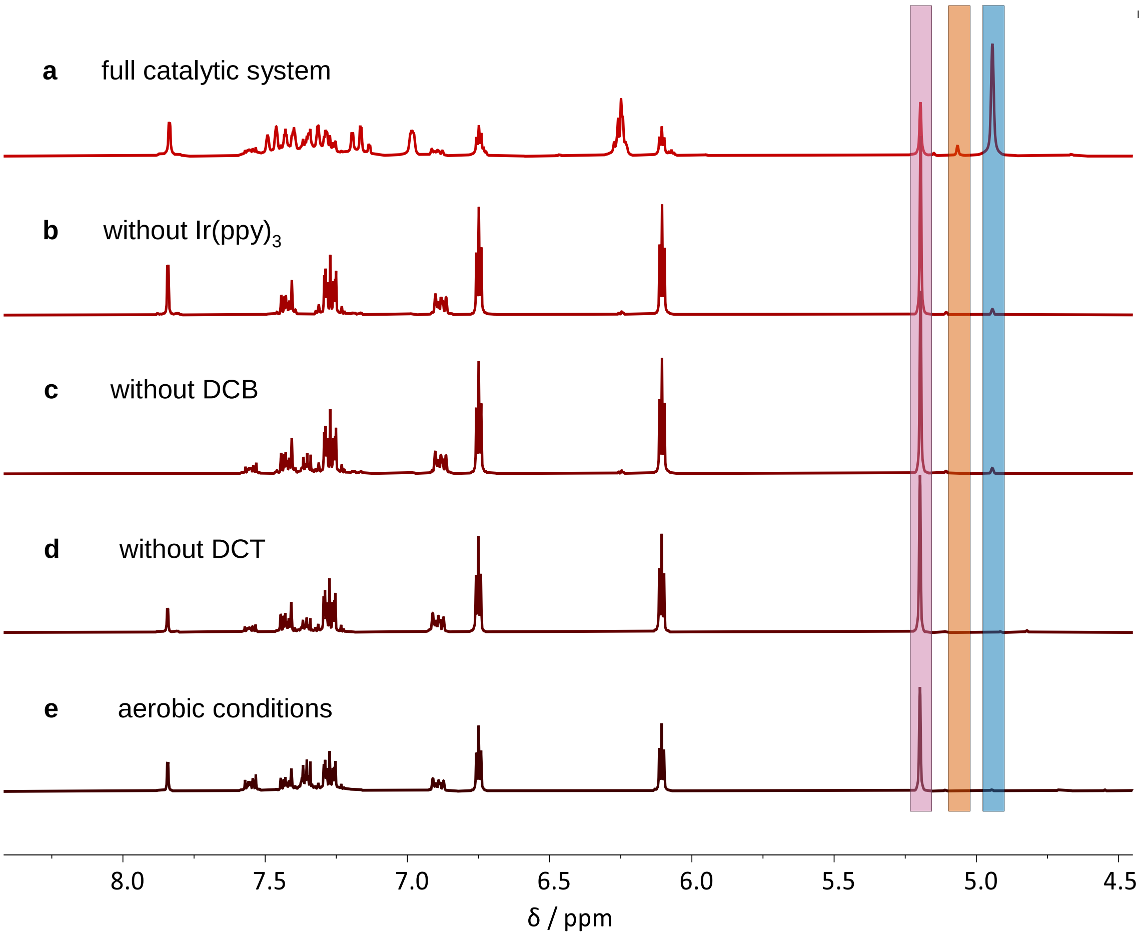


**Supplementary Fig. 13. a**, $^{1}$H NMR spectra monitoring the base-promoted homolytic aromatic substitution (BHAS) reaction of 1-(2-chlorobenzyl)-1H-pyrrole **1** (red background) to 5H-pyrrolo[2,1-a]isoindole **3** (blue background) after 21 hours of irradiation (using the same conditions as in Supporting Fig. 12), including the corresponding control experiments without $Ir(ppy)_3$ (**b**), without DCB (**c**), without TMP (**d**), and under aerobic conditions (**e**).

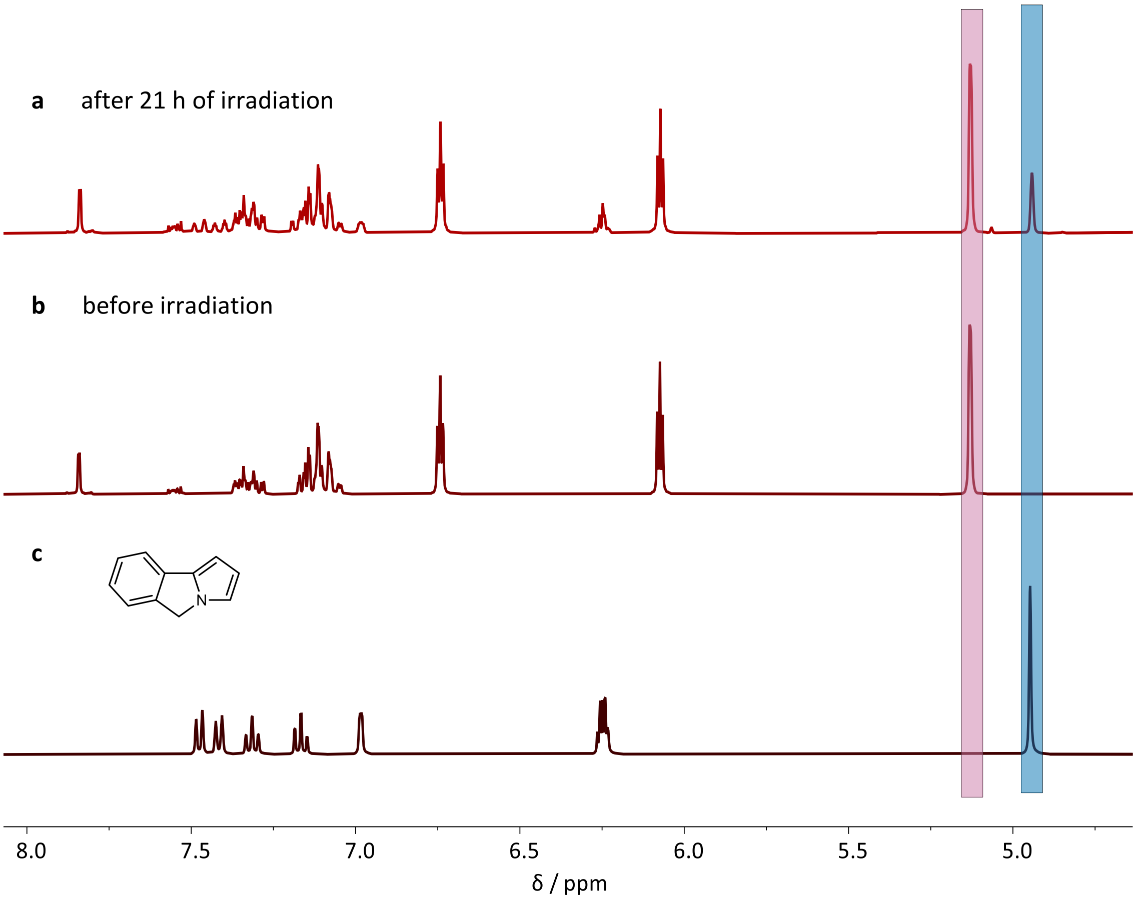


**Supplementary Fig. 14.** $^{1}$H NMR spectra monitoring the base-promoted homolytic aromatic substitution (BHAS) reaction of 1-(2-fluorobenzyl)-1H-pyrrole **2** (red background) to 5H-pyrrolo[2,1-a]isoindole **3** (blue background). The signal at 7.8 ppm originates from DCB. **a** & **b**, $^{1}$H NMR spectra of the reaction mixture containing 800 µM Ir(ppy)$_3$, 4 mM DCB, 80 mM TMA and 40 mM 1-(2-fluorobenzyl)-1H-pyrrole **2** in argon-saturated $CD_3CN$ before (**a**) and after 21 hours of 447 nm cw laser (2 W) irradiation (**b**). **c**, reference spectra of the desired product 5H-pyrrolo[2,1-a]isoindole **3**.

### Intra-molecular nucleophilic radical substitution reaction

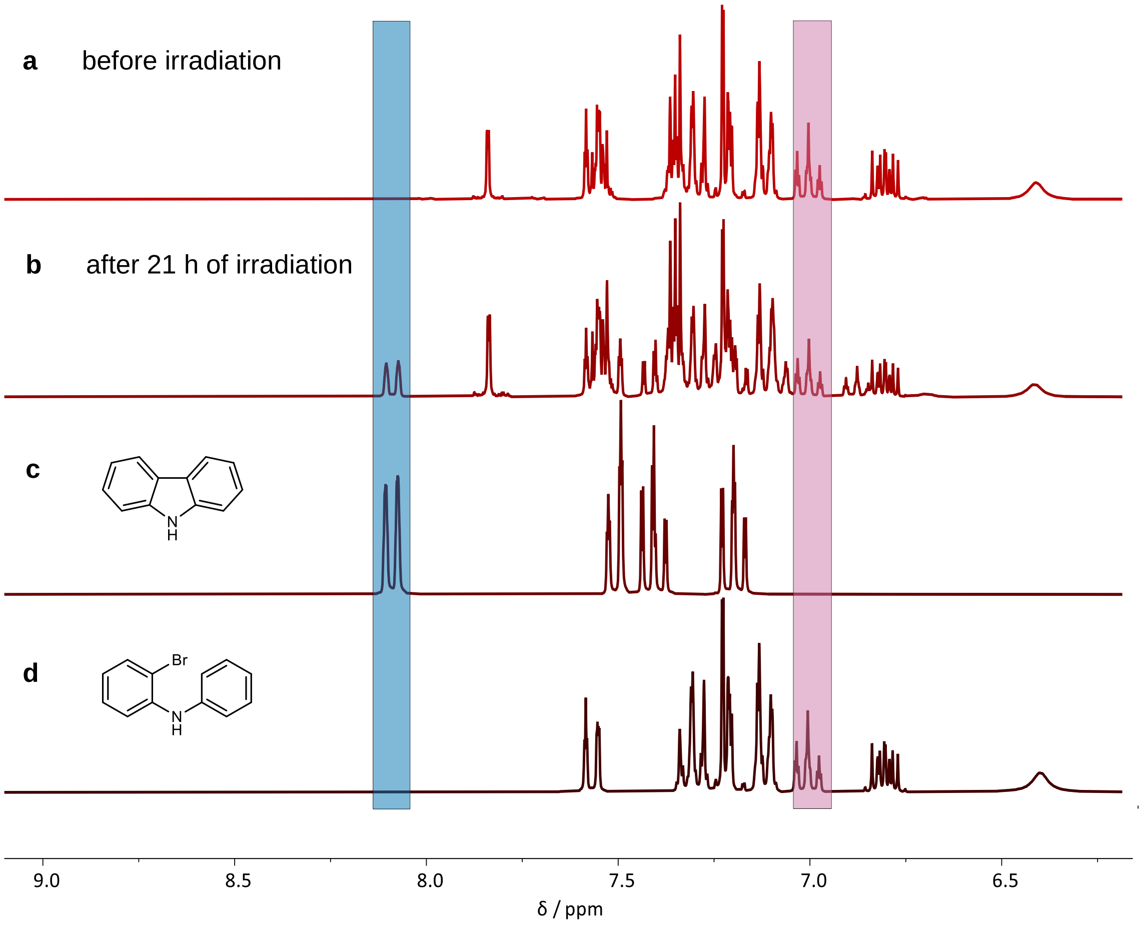


**Supplementary Fig. 15.** $^{1}$H NMR spectra monitoring the intra-molecular nucleophilic radical substitution reaction of 2-bromo-*N*-phenylaniline **4** (red background) to carbazole **7** (blue background). The signal at 7.8 ppm originates from DCB. **a** & **b**, $^{1}$H NMR spectra of the reaction mixture containing 800 μM Ir(ppy)$_3$, 4 mM DCB, 80 mM TMA, and 40 mM bromo-*N*-phenylaniline **4** in argon-saturated CD$_3$CN before (**a**) and after 21 hours of 447 nm cw laser (2 W) irradiation (**b**). **c** & **d**, Reference spectra of carbazole **7** (**c**) and the starting material bromo-*N*-phenylaniline **4** (**d**).

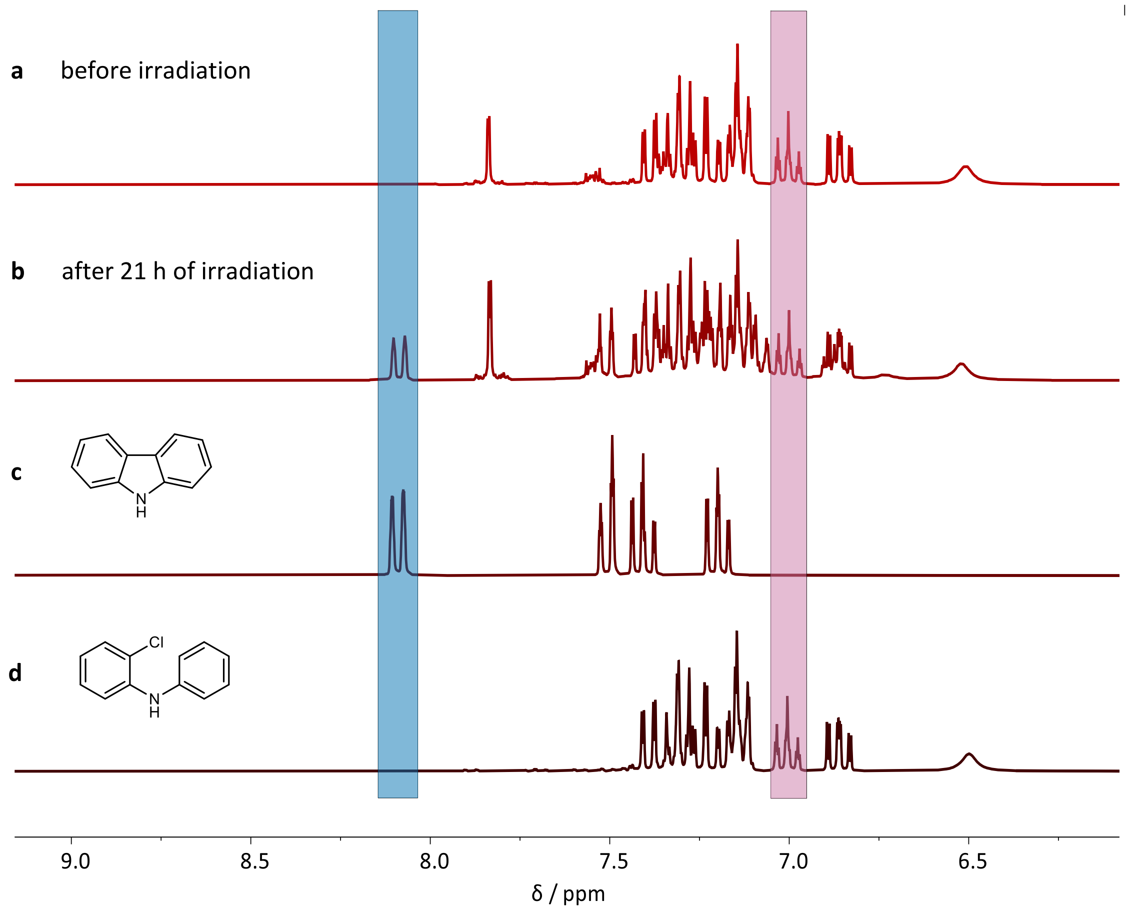


**Supplementary Fig. 16.** $^1$H NMR spectra monitoring the intra-molecular nucleophilic radical substitution reaction of 2-chloro-*N*-phenylaniline **5** (red background) to carbazole **7** (blue background). The signal at 7.8 ppm originates from DCB. **a** & **b**, $^1$H NMR spectra of the reaction mixture containing 800 µM Ir(ppy)$_3$, 4 mM DCB, 80 mM TMA, and 40 mM chloro-N-phenylaniline **5** in argon-saturated $CD_3CN$ before (**a**) and after 21 hours of 447 nm cw laser irradiation (**b**). **c** & **d**, Reference spectra of carbazole **7** (**c**) and the starting material chloro-*N*-phenylaniline **5** (**d**).

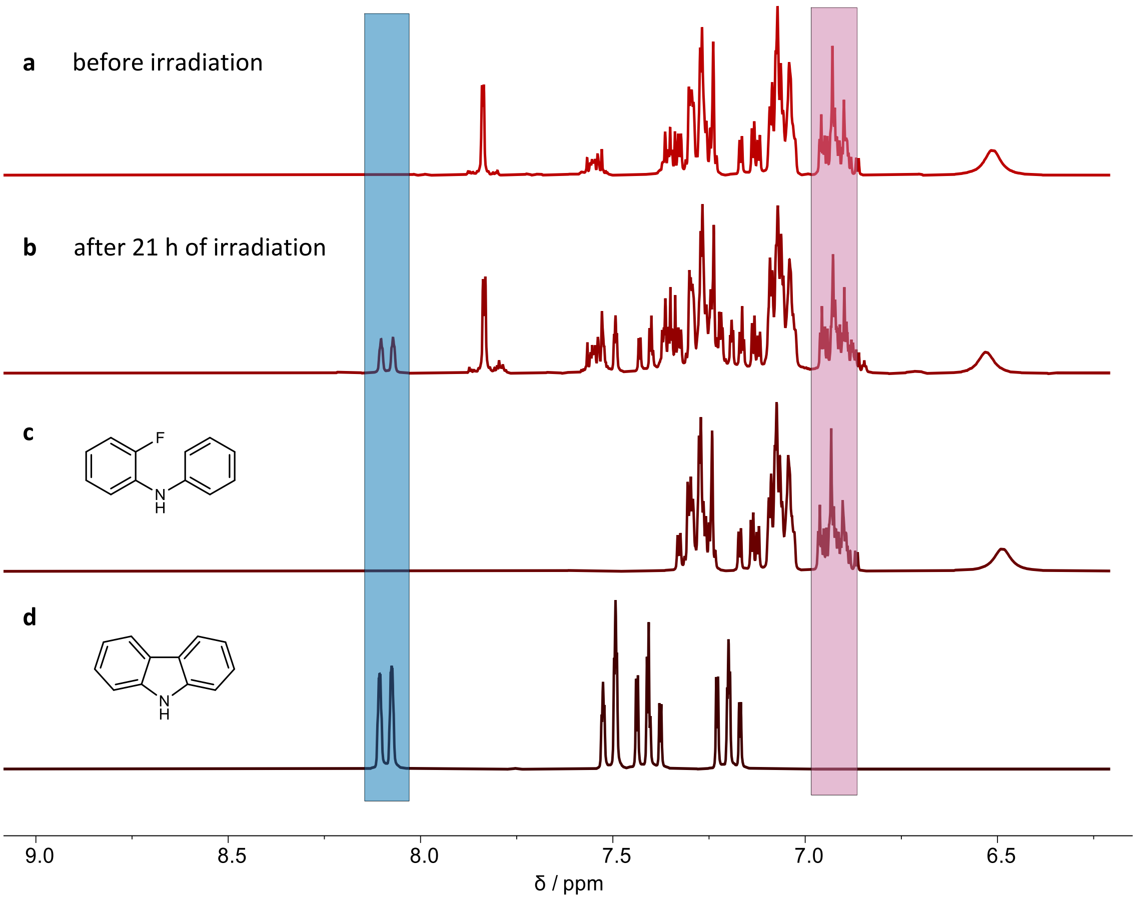


**Supplementary Fig. 17.** $^1$H NMR spectra monitoring the intra-molecular nucleophilic radical substitution reaction ($S_{RN}1$) of 2-fluoro-*N*-phenylaniline **6** (red background) to carbazole **7** (blue background). The signal at 7.8 ppm originates from DCB. **a** & **b**, $^1$H NMR spectra of the reaction mixture containing 800 µM Ir(ppy)$_3$, 4 mM DCB, 80 mM TMA, and 40 mM fluoro-*N*-phenylaniline **6** in argon-saturated $CD_3CN$ before (**a**) and after 21 hours of 447 nm cw laser (2 W) irradiation (**b**). **c** & **d**, Reference spectra of the starting material fluoro-*N*-phenylaniline (**c**) **6** and carbazole **7** (**d**).

## 5. Reaction quantum yield

The external reaction quantum yield ($\Phi_r$) is a key metric for evaluating reaction efficiency and is defined as the number of moles of photo-product formed ($n_{product}$) divided by the number of moles of emitted photons ($n_{photons}$) from the used light source (Supplementrary Equation (1)).[21]

$$\Phi_r = \frac{n_{\text{product}}}{n_{\text{photons}}} \qquad \text{Supplementrary Equation (1)}$$

The amount of photons emitted by the cw laser ($n_{photons}$) was determined with Supplementrary Equation (2), where $P_{laser}$ is the power output of the cw laser, $t$ is the irradiation time, $E_{photon}$ is the energy of single photons with a wavelength of 447 nm ($4.44 \times 10^{-19}$ J), and $N_A$ is Avogadro's constant.

$$n_{photons} = \frac{P_{\text{laser}} \times t}{E_{\text{photon}} \times N_A} \qquad \text{Supplementrary Equation (2)}$$

Using the amount of emitted laser photons ($n_{photons}$) incident on the sample and using the formed molar concentration of 5H-pyrrolo[2,1-a]isoindole product ($n_{product}$), the reaction quantum yield ($\Phi_r$) was calculated based on Supplementrary Equation (3). The results are shown in Fig. 4 in the main paper for different laser powers and variable reaction times ($t$).

$$\Phi_r = \frac{n_{\text{product}}}{n_{\text{photons}}} = \frac{n_{\text{product}}}{\frac{P_{\text{Laser}} \times t}{E_{\text{Photon}} \times N_A}} \qquad \text{Supplementrary Equation (3)}$$

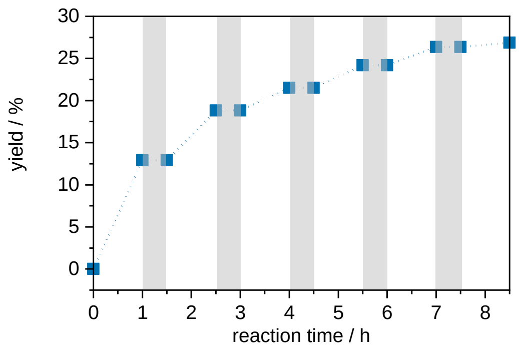


**Supplementary Fig. 18.** Product yield resulting from the BHAS reaction mixture containing 800 µM Ir(ppy)$_3$, 4 mM DCB, 40 mM substrate **1**, and 80 mM TMA in $CD_3CN$ under 447nm cw laser irradiation (1 W) by tracing the benzylic $^1$H NMR resonances of substrate **1** (1-(2-chlorobenzyl)-1H-pyrrole) at 5.2 ppm and product **3** (5H-pyrrolo[2,1-a]isoindole) at 4.9 ppm. The grey areas indicate the time periods during which no irradiation occurred.

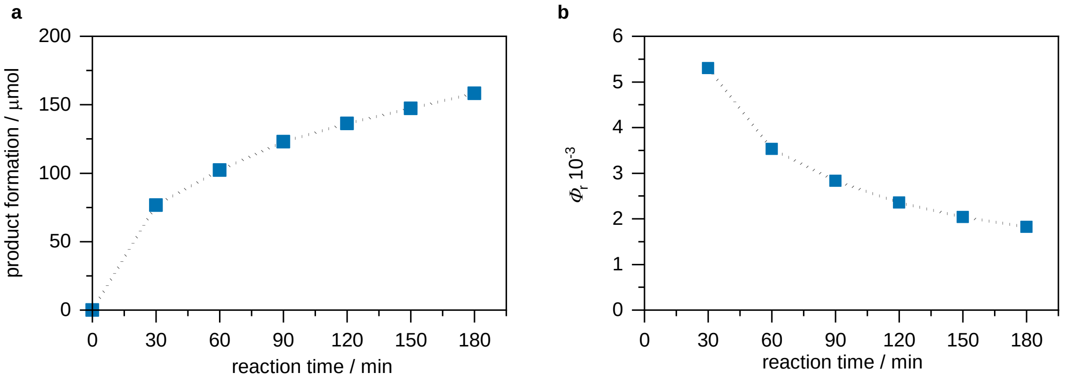


**Supplementary Fig. 19. a**, Product formation of the BHAS reaction containing 800 µM Ir(ppy)$_3$, 4 mM DCB, 40 mM substrate **1**, and 80 mM TMA in $CD_3CN$ under 447nm cw laser irradiation (2.13 W) by tracing the benzylic $^1$H NMR resonances of the substrate (1-(2-chlorobenzyl)-1H-pyrrole) at 5.2 ppm and the product (5H-pyrrolo[2,1-a]isoindole) at 4.9 ppm. Product formation was determined based on trimethyl(phenyl)silane as an internal standard. **b**, Resulting reaction quantum yield calculated with equation S3.

## 6. Ultrafast transient UV-Vis absorption spectroscopy

***Experimental setup***

Prior studies of related cyanoarene radical anion species used ultrafast two-color pump-pump-probe spectroscopy.[19,22,23] Adapting this methodology to the here investigated DCB and DCT compounds (Supplementary Fig. 20a), the first light pulse can excite their ground states, and in the presence of an electron donor, the radical anion ($DCX^{•-}$) is expected to be formed. After a time delay of several nanoseconds, a second pump pulse could in principle, excite the radical anion, followed by the probe pulse detecting the transient absorption signal of the photoproducts. However, such a two-color pump-pump-probe laser system with sub-picosecond time resolution was unavailable for our study. Alternative methods to generate the respective radical anions include electrochemical, photochemical, or chemical methods, followed by ultrafast pump-probe spectroscopy (Supplementary Fig. 20b). However, these methods to generate $DCX^{•-}$ proved unsuccessful in our hands due to irreproducible radical anion concentrations. According to a similar approach to a previous study,[19] continuous direct DCX irradiation during the ultrafast pump-probe experiment in the presence of an electron donor can, in principle, be used to establish a sizeable steady-state concentration of $DCX^{•-}$, enabling the observation of the excited-state dynamics of $^{2*}DCX^{•-}$ (Supplementary Fig. 20c). However, in the case of DCB and DCT, this approach would require UV light irradiation (< 350 nm), where continuous wave lasers were unavailable to us. Consequently, this methodology based on direct DCX excitation was not viable for this study. To overcome this technical challenge, $DCX^{•-}$ was generated through sensitization using the previously tested $Ir(ppy)_3$ photosensitizer, which absorbs in the visible range, combined with an ultrafast transient UV-Vis pump-probe experiment. Upon photoexcitation of an argon-saturated DMF solution containing 2 mM of $Ir(ppy)_3$ and 50 mM of DCB, photoinduced single electron transfer (SET) occurs, resulting in the formation of $DCB^{•-}$. The continuous wave laser beam was directed to the same area in the sample cuvette as the pump and probe pulse beams of the employed transient absorption spectroscopy setup. After continuous 405 nm irradiation during approximately 3 minutes, ultrafast transient UV-Vis pump-probe spectra were recorded (Supplementary Fig. S21), using 700 nm or 1200 nm excitation pulses, while keeping the 405-nm cw laser running. For all measurements, 0.1 M tetra-*n*-butylammonium dihydrogen phosphate was added, because bulky organic salts might be able to stabilize radical anions, to improve the overall performance of the continuous(pump)-pump-probe experiment.[19,20] In the case of DCT, photoexcitation of an argon-saturated DMF solution containing $Ir(ppy)_3$ (2 mM), DCB (50 mM), both SET and TTET occur in parallel, resulting in the simultaneous formation of $DCT^{•-}$ and $^{3*}DCT$ (see above).To increase the $DCT^{•-}$ concentration and to minimize the formation of $^{3*}DCT$, DIPEA was added as an electron donor for the continuous(pump)-pump-probe experiment.

***Discussion of kinetics for single electron transfer from $^{2*}DCB^{\bullet-}$ to the substrate***

In case the initial photoinduced SET step between $^{2*}DCB^{\bullet-}$ and substrate **1** would be governed by diffusion, the maximum achievable quantum yield ($\Phi_r$) for the BHAS reaction would be limited by the efficiency of this first SET process, assuming that each subsequent step occurs quantitatively.

Hence, the reaction efficiency ($\eta$) was estimated using Supplementrary Equation (4);

$$\eta = \frac{\tau_0 - \tau}{\tau_0} = \frac{k_q \times [Q]}{\tau_0^{-1} + k_q \times [Q]} \qquad \text{Supplementrary Equation (4)}$$

where $k_q$ is the diffusion-limited rate constant in $CH_3CN$ at 25 °C ($1.9 \times 10^{10}$ $M^{-1}$ $s^{-1}$), $\tau_0$ the natural $D_1$ excited state lifetime of $^{2*}DCB^{\bullet-}$ (4ps), and [Q] is the employed concentration of substrate **1** (40 mM) in the BHAS reaction. The resulting quenching efficiency of $3.2 \times 10^{-3}$ is significantly lower than the observed maximum reaction quantum yield of $5 \times 10^{-3}$, indicating pre-association between $^{2*}DCB^{\bullet-}$ and substrate **1**

***Discussion of the competition between bimolecular diffusion-controlled SET and SET in pre-aggregated adducts***

The pre-association between $^{2*}DCB^{\bullet-}$ and substrate **1** was probed by a two-color (continuous)pump-pump-probe experiment using various concentrations of **1** up to 130 mM. To rule out significant effects from diffusion-controlled bimolecular SET, the quenching efficiency was calculated using Supplementrary Equation (4), where $k_q$ is the diffusion-based rate constant in DMF ($7.6 \times 10^{9}$ $M^{-1}$ $s^{-1}$), $\tau_0$ the natural $D_1$ excited state lifetime of $^{2*}DCB^{\bullet-}$ (4ps), and [Q] is the employed concentration of substrate **1** (130 mM) in the experiment. The resulting diffusion controlled bimolecular SET can occur with an efficiency of less than 0.004 under the relevant conditions, too small to have a significant effect on the pre-association measurements.

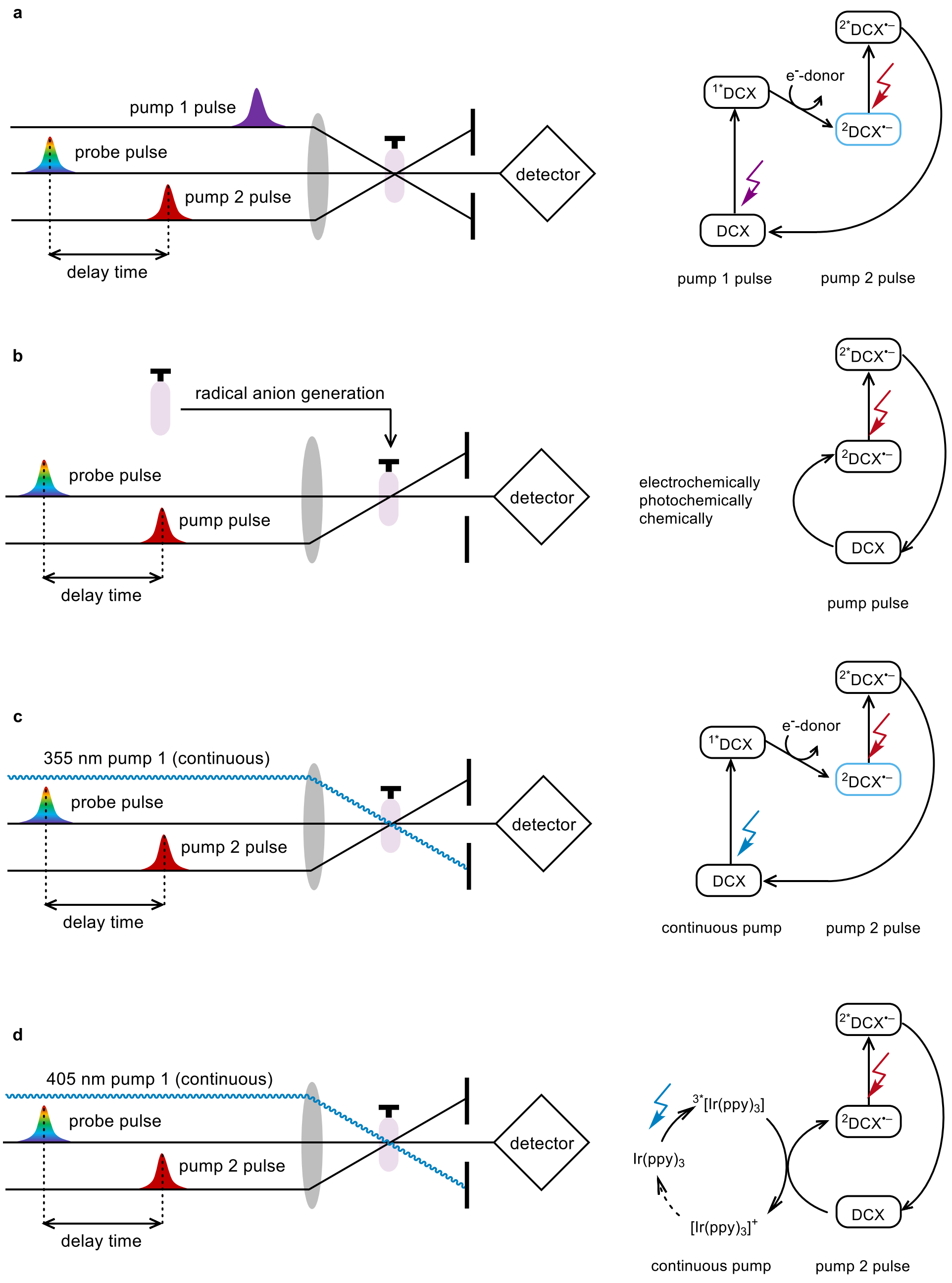


**Supplementary Fig. 20.** Different experimental approaches and possible strategies to investigate the excited-state dynamics of $DCB^{\bullet-}$ and $DCT^{\bullet-}$. **a**, Potential experimental setup for a sub-femtosecond two-color pump-pump-probe experiment. **b**, Potential experimental setup involving the active photocatalyst's electrochemical, photochemical, or chemical generation, followed by a sub-femtosecond pump-probe experiment. **c**, Potential experimental setup using a 355 nm continuous wave (cw) laser to generate a steady-state concentration of the active photocatalyst, followed by laser pulses of approximately 190 fs duration at 700 nm (for $DCB^{\bullet-}$) or 1200 nm (for $DCT^{\bullet-}$) and a white-light probe. **d**, Experimental setup used in this study, employing a 405 nm cw laser (0.5 W), a laser providing pulses of approximately 190 fs duration at 700 nm (for $DCB^{\bullet-}$) or 1200 nm (for $DCT^{\bullet-}$), and a white-light probe. For details, see the accompanying text.

## Excited state dynamics of $^{2*}DCB^{\bullet-}$ and $^{2*}DCT^{\bullet-}$

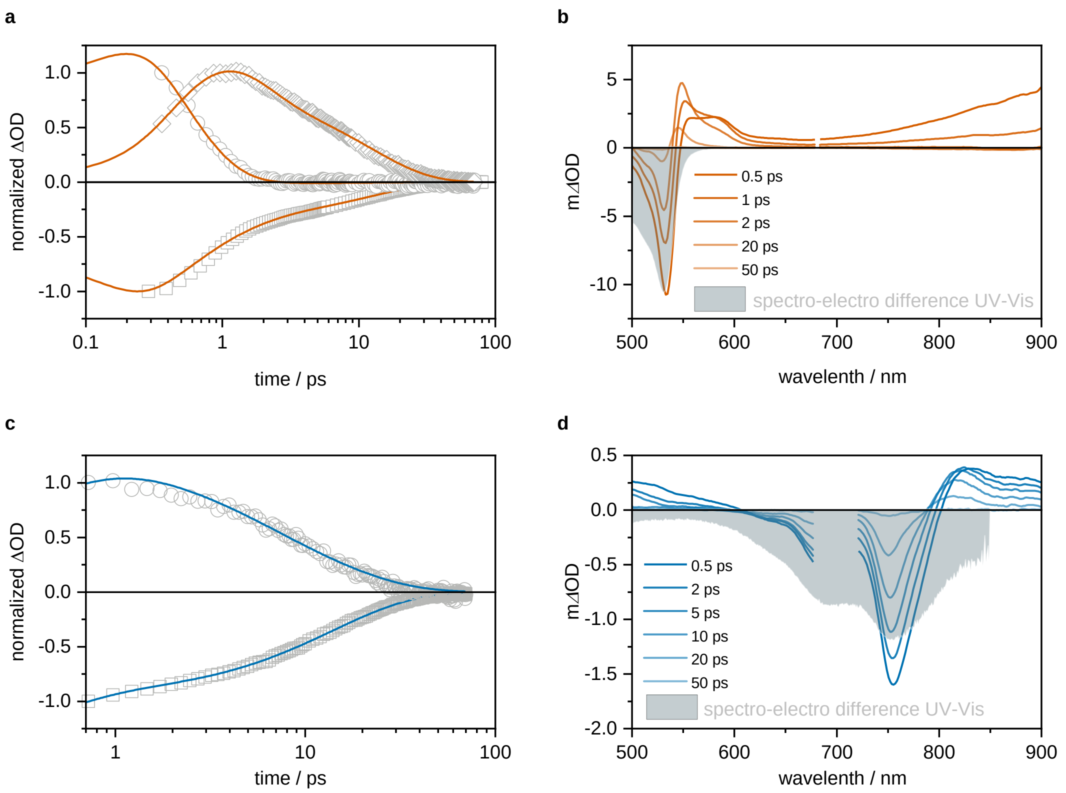


**Supplementary Fig. 21. a**, Decay profiles of the GSB signal at 544 nm (squares), the ESA at 565 nm (rhombuses), and 786 nm (circles) for $^{2*}DCT^{\bullet-}$, where the symbols represent the experimental data, and the orange traces represent the result of a global fit. **b**, Transient UV-Vis absorption spectra of $^{2*}DCT^{\bullet-}$ were recorded upon 1200 nm pulse excitation of a constantly irradiated (405 nm, 500 mW) argon-saturated DMF solution containing 2 mM $Ir(ppy)_3$, 50 mM DCT, 100 mM DIPEA, and 0.1 M tetra-*n*-butylammonium dihydrogenphosphate. **c**, Decay profile of the GSB signal at 757 nm (squares) and the ESA at 845 nm (circles) for $^{2*}DCB^{\bullet-}$, where the symbols represent the experimental data, and the blue traces represent the global fit. **d**, Transient UV-Vis absorption spectra of $^{2*}DCB^{\bullet-}$ were recorded upon 700 nm pulse excitation of a constantly irradiated (405 nm, 500 mW) argon-saturated DMF solution containing 2 mM $Ir(ppy)_3$, 50 mM DCT, and 0.1 M tetra-*n*-butylammonium dihydrogenphosphate. The grey areas in **b** and **d** mark the negative UV-Vis changes on electrochemical reduction at −1.5 V versus an Ag pseudo reference electrode of an argon-saturated DMF solution containing $TBAPF_6$ (0.1 M) as a supporting electrolyte and 600 μM DCT (**b**) or 600 μM DCB (**d**).

## 7. NMR spectra

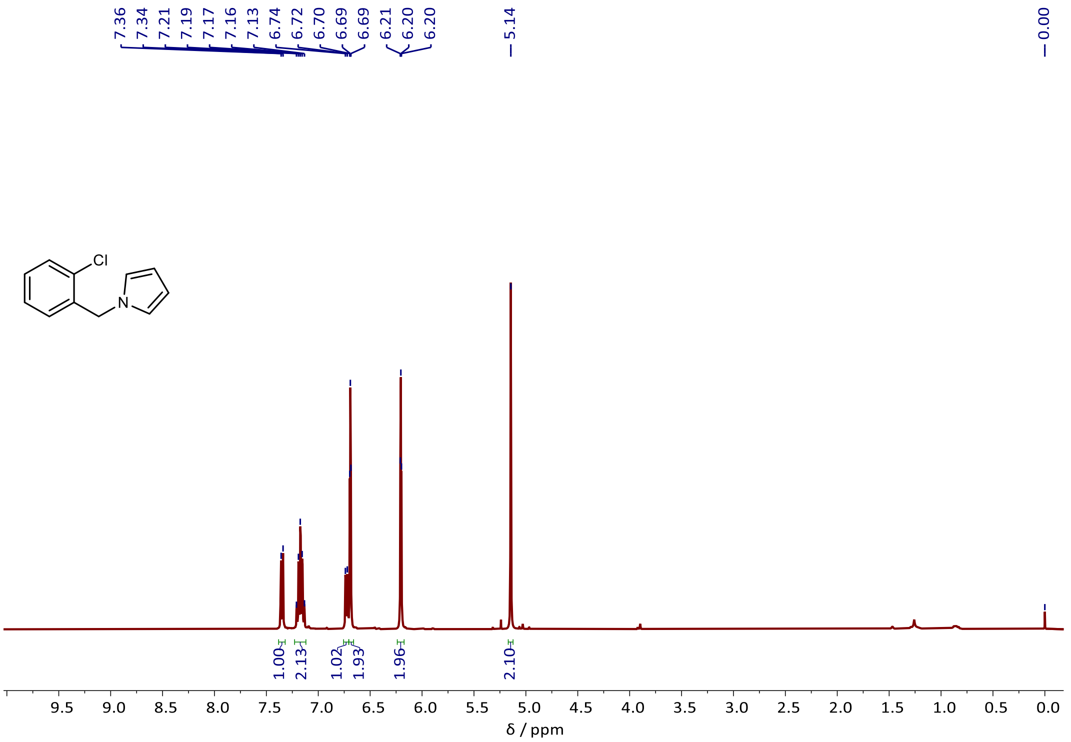


***Supplementary Fig. 22.*** *$^{1}$H NMR spectrum of compound **1** (400 MHz, $CDCl_3$).*

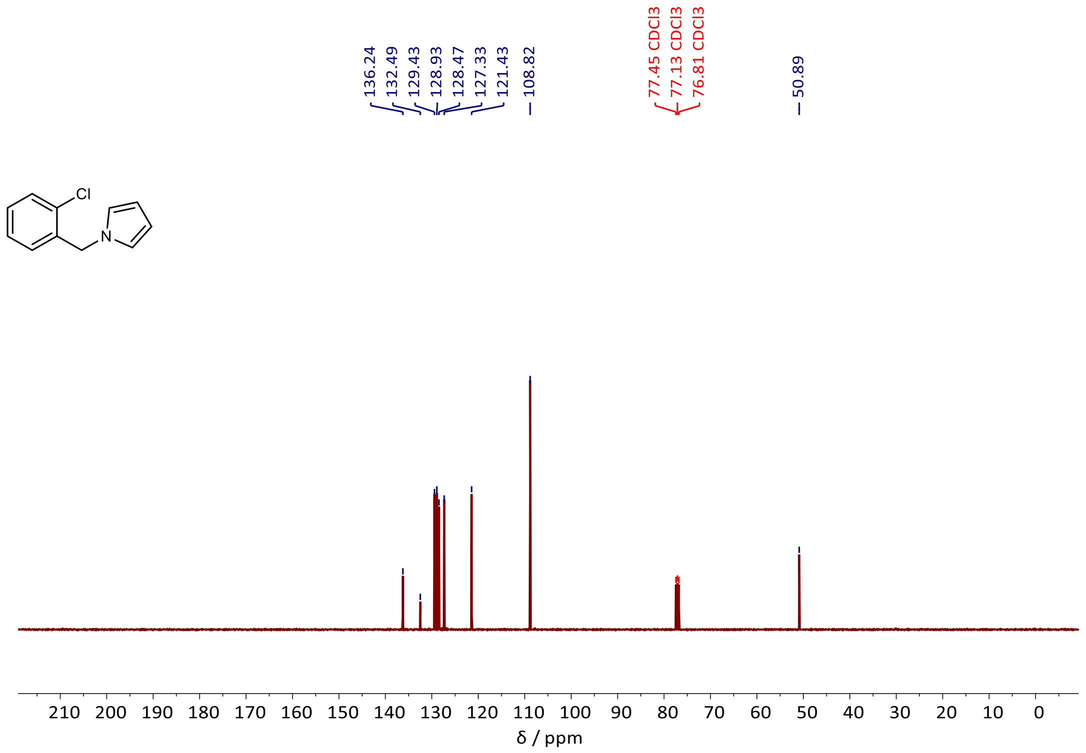


***Supplementary Fig. 23.*** *$^{13}$C NMR spectrum of compound **1** (125 MHz, $CDCl_3$).*

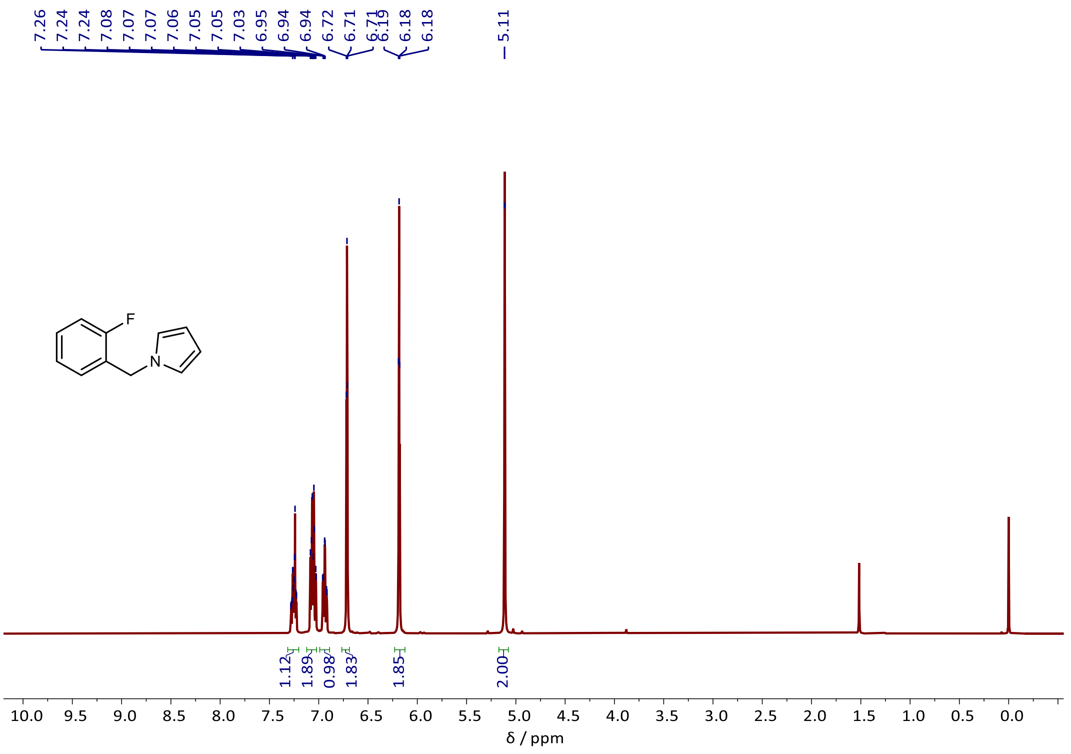

**Supplementary Fig. 24.** *$^{1}H$ NMR spectrum of compound* ***2*** *(400 MHz, $CDCl_3$).*

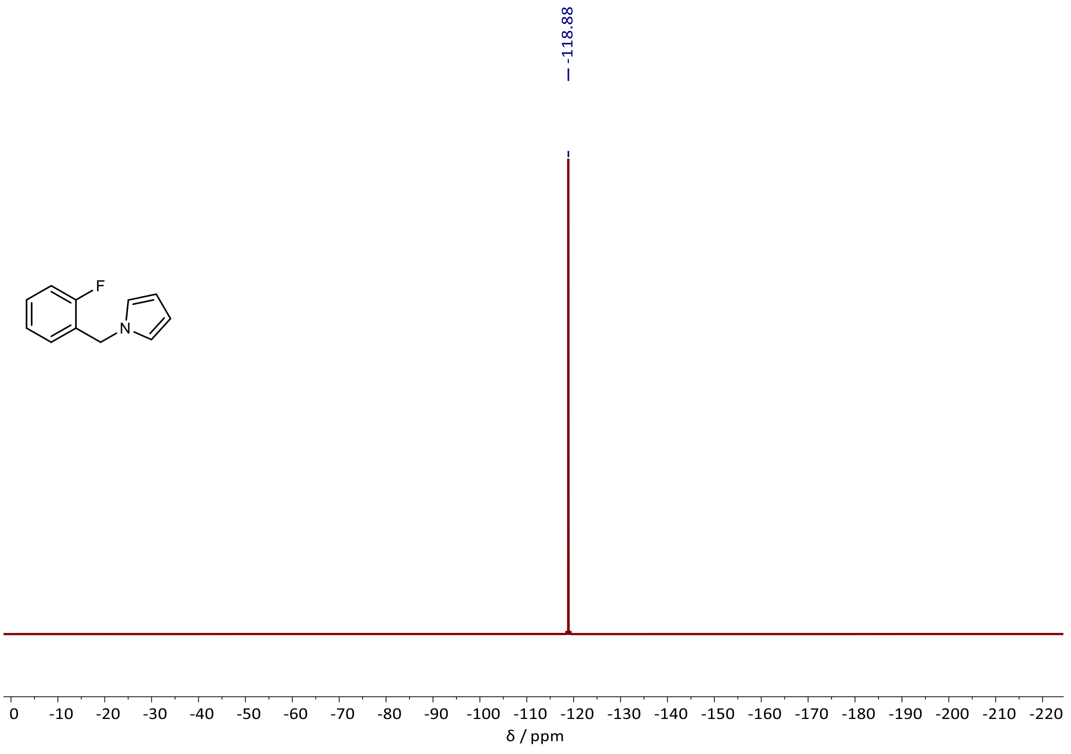

**Supplementary Fig. 25.** *$^{19}F$ NMR spectrum of compound* ***2*** *(376 MHz, $CDCl_3$).*

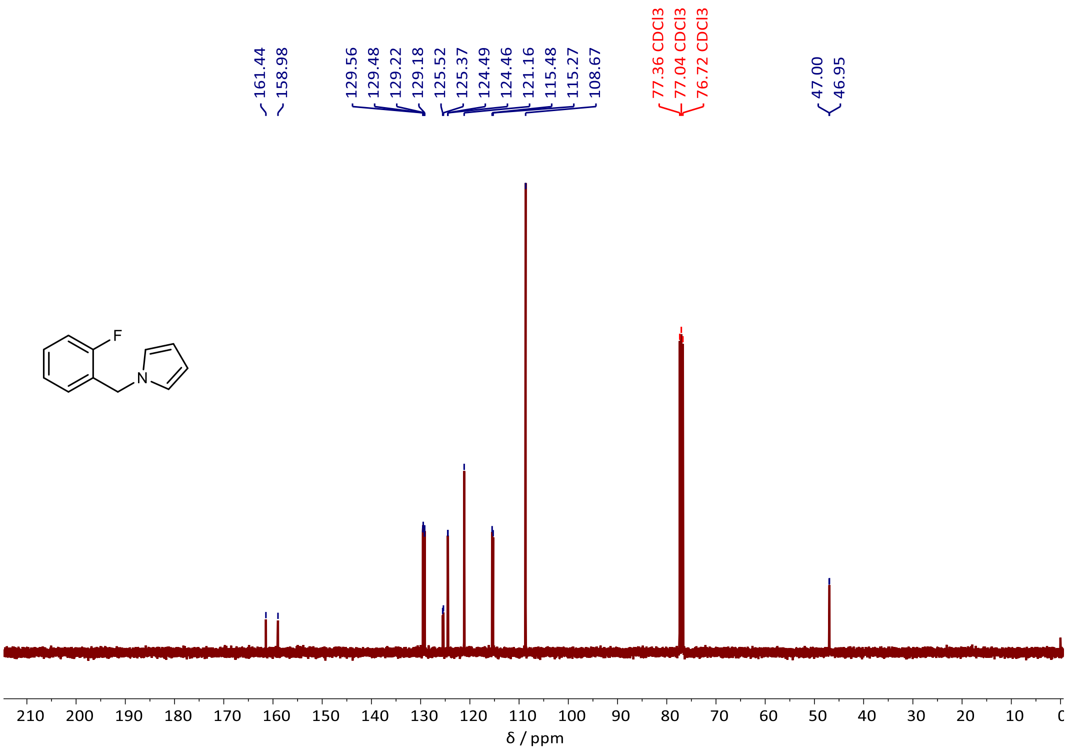


***Supplementary Fig. 26.*** *$^{13}$C NMR spectrum of compound* ***2*** *(125 MHz, $CDCl_3$).*

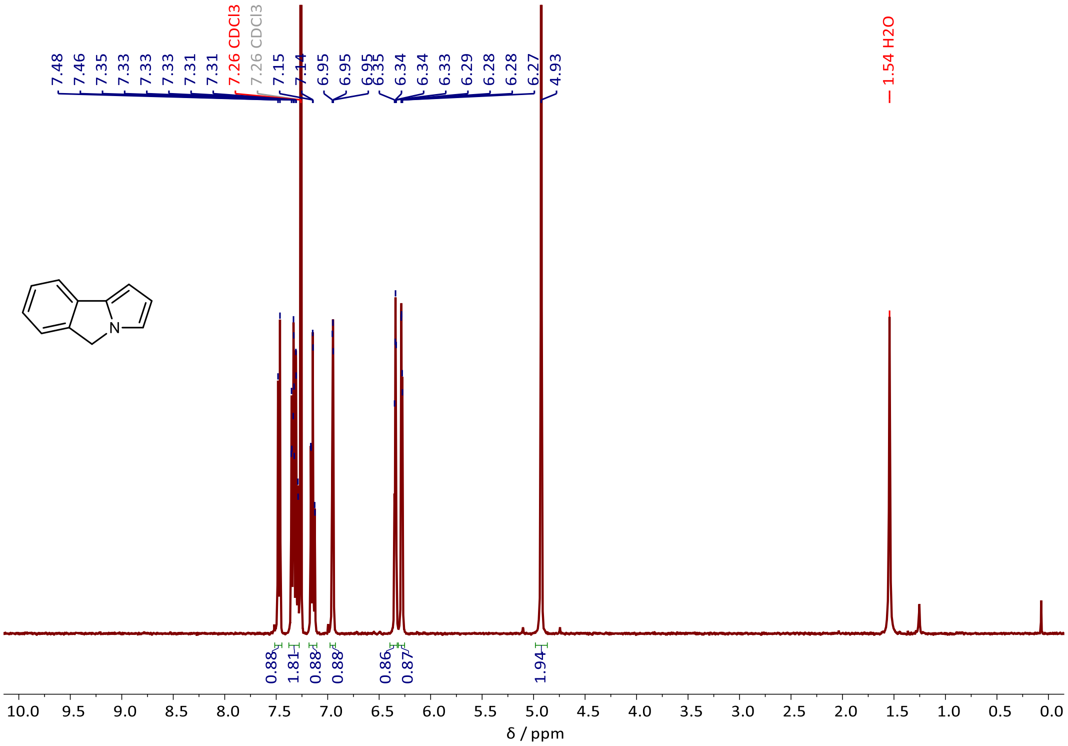


***Supplementary Fig. 27.*** *$^1$H NMR spectrum of compound* ***3*** *(400 MHz, $CDCl_3$).*

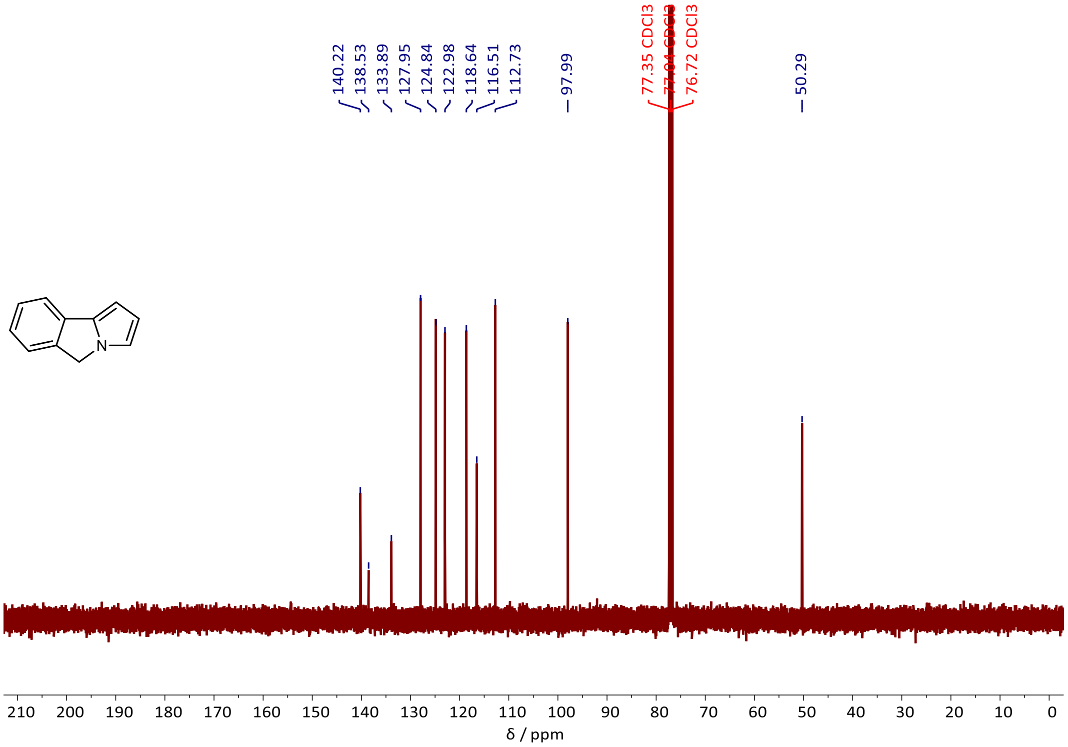


***Supplementary Fig. 28.*** *$^{13}$C NMR spectrum of compound* ***3*** *(125 MHz, $CDCl_3$).*

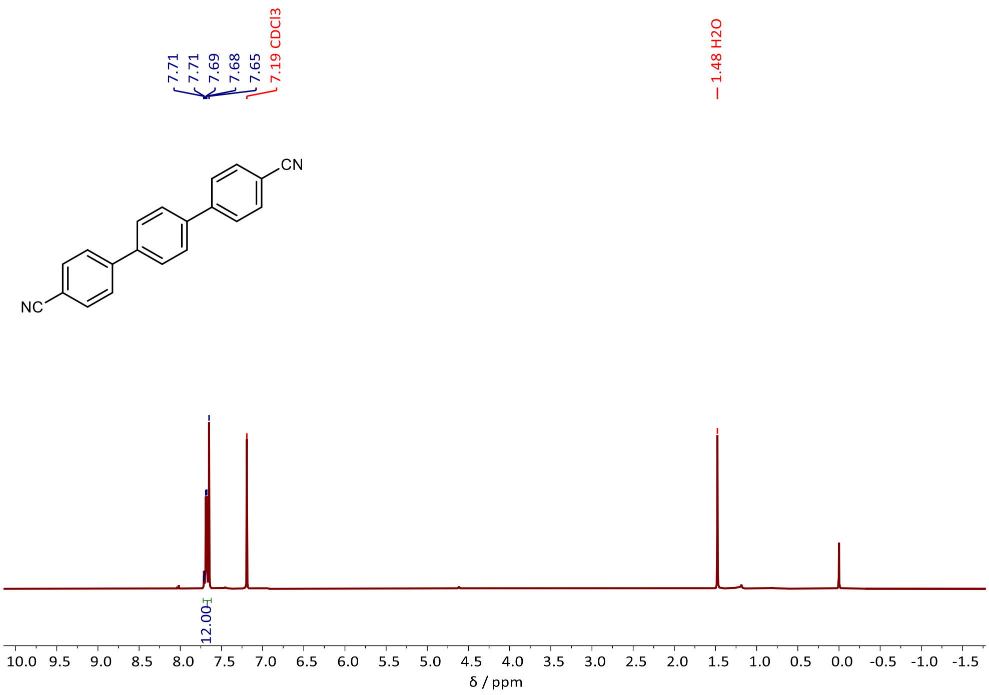


***Supplementary Fig. 29.*** *$^{1}H$ NMR spectrum of DCT (400 MHz, $CDCl_3$).*

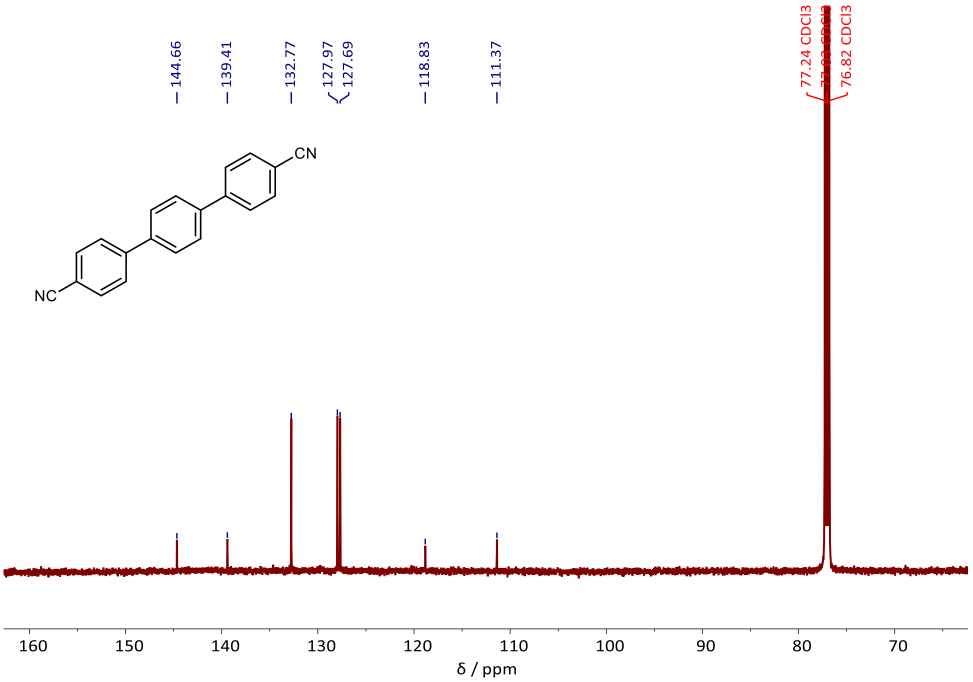


***Supplementary Fig. 30.*** *$^{13}C$ NMR spectrum of DCT (600 MHz, $CDCl_3$).*

## 8. References


1 Fulmer, G. R. *et al.* NMR Chemical Shifts of Trace Impurities: Common Laboratory Solvents, Organics, and Gases in Deuterated Solvents Relevant to the Organometallic Chemist. *Organometallics* **29**, 2176-2179 (2010). https://doi.org/10.1021/om100106e

2 Kerzig, C., Guo, X. & Wenger, O. S. Unexpected Hydrated Electron Source for Preparative Visible-Light Driven Photoredox Catalysis. *J. Am. Chem. Soc.* **141**, 2122-2127 (2019). https://doi.org/10.1021/jacs.8b12223

3 Kuss-Petermann, M. & Wenger, O. S. Pump-Pump-Probe Spectroscopy of a Molecular Triad Monitoring Detrimental Processes for Photoinduced Charge Accumulation. *Helv. Chim. Acta* **100**, e1600283 (2017). https://doi.org/10.1002/hlca.201600283

4 Deb, I., Coiro, D. J. & Seidel, D. Decarboxylative formation of *N*-alkyl pyrroles from 4-hydroxyproline. *Chem. Commun.* **47**, 6473-6475 (2011). https://doi.org/10.1039/c1cc11560j

5 Vijay Kumar, A. & Rama Rao, K. trans-4-Hydroxy-*L*-proline: a novel starting material for *N*-alkylpyrroles synthesis. *Tetrahedron Lett.* **52**, 3237-3239 (2011). https://doi.org/10.1016/j.tetlet.2011.04.045

6 Jang, J. H. *et al.* Synthesis of Redox-Active Phenanthrene-Fused Heteroarenes by Palladium-Catalyzed C-H Annulation. *Org. Lett.* **22**, 1280-1285 (2020). https://doi.org/10.1021/acs.orglett.9b04545

7 Ismail, M. A. *et al.* Synthesis, DNA Affinity, and Antiproton Activity of Linear Dications: Terphenyl Diamidines and Analogues. *J. Med. Chem.* **49**, 5324-5332 (2006). https://doi.org/10.1021/jm060470p

8 Moneo, Á., Carvalho, M. F. N. N. & Telo, J. P. Dicyanoaromatic radical anions as mixed valence species. *J. Phys. Org. Chem.* **25**, 559-565 (2012). https://doi.org/10.1002/poc.2905

9 Glaser, F. & Wenger, O. S. Red Light-Based Dual Photoredox Strategy Resembling the Z-Scheme of Natural Photosynthesis. *JACS Au* **2**, 1488-1503 (2022). https://doi.org/10.1021/jacsau.2c00265

10 Rehm, D. & Weller, A. Kinetics of fluorescence quenching by electron and H-atom transfer. *Isr. J. Chem.* **8**, 259-271 (1970). https://doi.org/10.1002/ijch.197000029

11 Rehm, D. & Weller, A. Kinetik und Mechanismus der Elektronübertragung bei der Fluoreszenzlöschung in Acetonitril. *Ber. Bunsen-Ges. Phys. Chem.* **73**, 834– 839 (1969). https://doi.org/10.1002/bbpc.19690730818

12 Day, J. I., Teegardin, K., Weaver, J. & Chan, J. Advances in Photocatalysis: A Microreview of Visible Light Mediated Ruthenium and Iridium Catalyzed Organic

Transformations. *Org. Process. Res. Dev.* **20**, 1156-1163 (2016). https://doi.org/10.1021/acs.oprd.6b00101

13 Pfund, B. *et al.* UV Light Generation and Challenging Photoreactions Enabled by Upconversion in Water. *J. Am. Chem. Soc.* **142**, 10468–10476 (2020). https://doi.org/10.1021/jacs.0c02835

14 Schreier, M. R. *et al.* Water-Soluble Tris(cyclometalated) Iridium(III) Complexes for Aqueous Electron and Energy Transfer Photochemistry. *Acc. Chem. Res.* **55**, 1290-1300 (2022). https://doi.org/10.1021/acs.accounts.2c00075

15 Schreier, M. R., Pfund, B., Steffen, D. M. & Wenger, O. S. Photocatalytic Regeneration of a Nicotinamide Adenine Nucleotide Mimic with Water-Soluble Iridium(III) Complexes. *Inorg. Chem.* **62**, 7636–7643 (2023). https://doi.org/10.1021/acs.inorgchem.2c03100

16 Maji, A., Singh, A., Mohanty, A., Maji, P. K. & Ghosh, K. Ferrocenyl palladacycles derived from unsymmetrical pincer-type ligands: evidence of Pd(0) nanoparticle generation during the Suzuki-Miyaura reaction and applications in the direct arylation of thiazoles and isoxazoles. *Dalton Trans.* **48**, 17083-17096 (2019). https://doi.org/10.1039/C9DT03465J

17 Pearson, R. M., Lim, C. H., McCarthy, B. G., Musgrave, C. B. & Miyake, G. M. Organocatalyzed Atom Transfer Radical Polymerization Using *N*-Aryl Phenoxazines as Photoredox Catalysts. *J. Am. Chem. Soc.* **138**, 11399-11407 (2016). https://doi.org/10.1021/jacs.6b08068

18 Speckmeier, E., Fischer, T. G. & Zeitler, K. A Toolbox Approach To Construct Broadly Applicable Metal-Free Catalysts for Photoredox Chemistry: Deliberate Tuning of Redox Potentials and Importance of Halogens in Donor-Acceptor Cyanoarenes. *J. Am. Chem. Soc.* **140**, 15353-15365 (2018). https://doi.org/10.1021/jacs.8b08933

19 Beckwith, J. S., Aster, A. & Vauthey, E. The excited-state dynamics of the radical anions of cyanoanthracenes. *Phys. Chem. Chem. Phys.* **24**, 568–577 (2022). https://doi.org/10.1039/d1cp04014f

20 Kellett, M. A. & Whitten, D. G. Surprising Differences in the Reactivity of Cyanoaromatic Radical Anions Generated by Photoinduced Electron Transfer. *J. Am. Chem. Soc.* **113**, 358-359 (1991). https://doi.org/10.1021/ja00001a052

21 Talbott, E. D., Burnett, N. L. & Swierk, J. R. Mechanistic and kinetic studies of visible light photoredox reactions. *Chem. Phys. Rev.* **4**, 031312 (2023). https://doi.org/10.1063/5.0156850

22 Beckwith, J. S., Lang, B., Grilj, J. & Vauthey, E. Ion-Pair Dynamics upon Photoinduced Electron Transfer Monitored by Pump-Pump-Probe Spectroscopy. *J. Phys. Chem. Lett.* **10**, 3688-3693 (2019). https://doi.org/10.1021/acs.jpclett.9b01431

23 Rieth, A. J., Gonzalez, M. I., Kudisch, B., Nava, M. & Nocera, D. G. How Radical Are "Radical" Photocatalysts? A Closed-Shell Meisenheimer Complex Is Identified as a Super-Reducing Photoreagent. *J. Am. Chem. Soc.* **143**, 14352–14359 (2021). https://doi.org/10.1021/jacs.1c06844
